# Sin3a associated protein 130 kDa, sap130, plays an evolutionary conserved role in zebrafish heart development

**DOI:** 10.3389/fcell.2023.1197109

**Published:** 2023-08-30

**Authors:** Ricardo A. DeMoya, Rachel E. Forman-Rubinsky, Deon Fontaine, Joseph Shin, Simon C. Watkins, Cecilia W. Lo, Michael Tsang

**Affiliations:** ^1^ Department of Developmental Biology, University of Pittsburgh School of Medicine, Pittsburgh, PA, United States; ^2^ Department of Cell Biology, University of Pittsburgh School of Medicine, Pittsburgh, PA, United States

**Keywords:** cardiac development, second heart field, SIN3A/HDAC complex, congenital heart disease, zebrafish

## Abstract

Hypoplastic left heart syndrome (HLHS) is a congenital heart disease where the left ventricle is reduced in size. A forward genetic screen in mice identified SIN3A associated protein 130 kDa (*Sap130*), part of the chromatin modifying SIN3A/HDAC complex, as a gene contributing to the etiology of HLHS. Here, we report the role of zebrafish *sap130* genes in heart development. Loss of *sap130a,* one of two *Sap130* orthologs, resulted in smaller ventricle size, a phenotype reminiscent to the hypoplastic left ventricle in mice. While cardiac progenitors were normal during somitogenesis, diminution of the ventricle size suggest the Second Heart Field (SHF) was the source of the defect. To explore the role of *sap130a* in gene regulation, transcriptome profiling was performed after the heart tube formation to identify candidate pathways and genes responsible for the small ventricle phenotype. Genes involved in cardiac differentiation and cardiac function were dysregulated in *sap130a*, but not in *sap130b* mutants. Confocal light sheet analysis measured deficits in cardiac output in *MZsap130a* supporting the notion that cardiomyocyte maturation was disrupted. Lineage tracing experiments revealed a significant reduction of SHF cells in the ventricle that resulted in increased outflow tract size. These data suggest that *sap130a* is involved in cardiogenesis via regulating the accretion of SHF cells to the growing ventricle and in their subsequent maturation for cardiac function. Further, genetic studies revealed an interaction between *hdac1* and *sap130a*, in the incidence of small ventricles. These studies highlight the conserved role of Sap130a and Hdac1 in zebrafish cardiogenesis.

## Introduction

Congenital heart diseases (CHDs) affect approximately 1% of live births per year and causes have been attributed to environmental and genetic factors (Nora, 1968; Fahed et al., 2013; Costain et al., 2016). Hypoplastic left heart syndrome (HLHS) is a critical CHD characterized by a reduced volume in the left ventricle and aortic and valve malformations (Connor and Thiagarajan, 2007; Barron et al., 2009). The genetic etiology of HLHS is complex and genetically heterogenous. Mouse models of HLHS were recovered from a large-scale mutagenesis screen (Liu et al., 2017), and among 8 lines, the *Ohia* mutant line was identified to have a digenic etiology for HLHS. This is comprised of mutations in SIN3A associated protein 130 kDa (SAP130) and protocadherin 9 (PCDHA9) that together causes HLHS comprising hypoplasia of all left-sided heart structures including the ventricle, aorta/aortic valve, and mitral valve. In pigs a CRISPR generated SAP130 allele caused embryonic lethality and tricuspid dysplasia and atresia, indicating SAP130 involvement in cardiac development in higher vertebrates (Gabriel et al., 2021). In zebrafish, maternal zygotic *sap130a* (*MZsap130a*) mutants resulted in a diminutive ventricle by 72 h post fertilization (hpf), confirming that SAP130 retains a conserved function among vertebrates during heart development (Liu et al., 2017; Gabriel et al., 2021).

SAP130 was identified as an interacting protein in the SIN3A complex, binding both SIN3A and Histone Deacetylase 1 (HDAC1), thought to stabilize the complex. It was theorized that the SAP130 C-terminus functioned as a transcriptional repressor in association with the SIN3A complex, while the N-terminus paradoxically could function as an activator (Fleischer et al., 2003). A knock-out allele of SAP130 in mice is peri-implantation lethal, unlike global knockouts of HDAC1 and SIN3A which die at later stages of development (Lagger et al., 2002; Dannenberg et al., 2005; Liu et al., 2017). These suggest multiple roles and stages of development where SAP130/SIN3A/HDAC1 are critical for life. SIN3A and HDACs epigenetically regulate transcription through histone and non-histone deacetylation events and are classically associated with gene repression. However, some studies have shown this complex to be a transcriptional activator in other contexts (Han et al., 2011; Kadamb et al., 2013; Adams et al., 2018). HDACs have been reported to regulate many aspects of development, including cardiac development in zebrafish, mouse, and chick models, as evidenced by treatment with a pan HDAC small molecule inhibitor, Trichostatin A (Hargreaves and Crabtree, 2011; McKinsey, 2011; Martinez et al., 2015). Zebrafish studies have revealed that *hdac1* is involved in Second Heart Field (SHF) development and in adult cardiac regeneration (Song et al., 2019; Buhler et al., 2021). In zebrafish, *hdac1* mutants have less cardiomyocytes (CMs) in the ventricle while inhibition of *hdac1* (and other class I HDACs) reveal reduced proliferation during regenerative events (Montgomery et al., 2007; Nambiar et al., 2007; Song et al., 2019; Buhler et al., 2021). Zebrafish *hdac1* mutants are embryonic lethal, similar to the mouse models, but *MZsap130a* mutants are viable as adults suggesting that *hdac1* and *sap130a* may have distinct functions in zebrafish cardiogenesis.

In addition to the Sin3a/Hdac1 complex, related chromatin modifying complexes like the BAF complex, have been shown to be involved in cardiogenesis (Lickert et al., 2004; Wang et al., 2004; Stankunas et al., 2008; Hang et al., 2010; Hargreaves and Crabtree, 2011; Takeuchi et al., 2011; Lei et al., 2012; Singh and Archer, 2014; Nakamura et al., 2016; Xiao et al., 2016; Sun et al., 2018; Alfert et al., 2019; Hota et al., 2019; Lei et al., 2019; Chen et al., 2022; Auman et al., 2023). A study describing the loss of *smarcc1a*, a BAF chromatin remodeling complex protein, in zebrafish resulted in dysmorphic cardiac chambers further highlighting the importance of chromatin remodeling in proper heart formation (Auman et al., 2023). Another part of the BAF complex in zebrafish *brg1*, when mutated reveals a reduction in CM proliferation leading to a smaller ventricle after 28hpf. The *brg1* mutants reveal changes in a working myocardium marker *nppa*, similar to mouse Brg1 mutants (Takeuchi et al., 2011). Other types of epigenetic regulation such as methylation are shown to be paired with chromatin remodeling events and are involved in cardiogenic processes (Carrozza et al., 2005; Joshi and Struhl, 2005; Keogh et al., 2005; Brown et al., 2006; Donlin et al., 2012; Voelkel et al., 2013; Singh and Archer, 2014; Xiao et al., 2018; Zhu et al., 2018; Bisserier et al., 2021). SET and MYND domain-containing lysine methyltransferase 4 (*smyd4*) mutants also result in reduced ventricle size in zebrafish and mouse, suggesting there is a common requirement of gene regulation for specifying heart organ size in vertebrates (Trotter and Archer, 2008). RNA sequencing (RNA-seq) analysis of *smyd4* zebrafish mutants revealed dysregulation of cardiac muscle contraction and metabolism genes. Moreover, cell culture studies revealed human SMYD4 and HDAC1 interact, further supporting a central requirement for *hdac1* in zebrafish cardiogenesis (Xiao et al., 2018). Taken together these suggest a potential epigenetic role for *sap130a* during development as part of the Sin3a complex.

Here we investigate the role of *sap130* genes in zebrafish by studying mutations in both *sap130a* and *sap130b*. Transcriptome profiling of 36hpf *MZsap130a* mutants revealed over 5,000 genes to be differentially expressed, including genes involved in the cardiac development and function. In genetic studies, an increase in embryos with small ventricles (SVs) were noted in *MZsap130a* embryos that were also heterozygous for *hdac1*. Furthermore, *MZsin3ab* mutants exhibit a SV phenotype at 48hpf. Collectively, these studies suggest a role for *sin3ab*/*hdac1*/*sap130a* in the SHF during zebrafish cardiogenesis.

## Materials and methods

### Zebrafish husbandry

All zebrafish experiments and protocols were performed according to protocols approved by the Institutional Animal Care and Use Committee (IACUC) at the University of Pittsburgh in agreement with NIH guidelines. Wild-type AB*, *Tg*(*myl7:GFP*)^*twu34*^ (Huang et al., 2003), *Tg*(*nkx2.5:kaeda*)^*fb9*^ (Guner-Ataman et al., 2013), *sap130a* ^*pt32a*^ (Liu et al., 2017), *hdac1* ^*b382*^ (Ignatius et al., 2013).

Adult tail fin clips or whole embryos for genotyping assays was performed as previously described (Jing, 2012). Restriction fragment length polymorphism (RFLP) genotyping for *sap130a* ^*pt32a*^, *sap130b* ^*pt35b*^, *sin3ab* ^*pt36a*^ and *hdac1* ^*b382*^ used the primers and enzymes listed in Supplementary Table S1.

### CRISPR/Cas9 mutant allele generation

The CRISPR/Cas9 protocol (Gagnon et al., 2014) was used to establish mutant lines. This protocol used Sp6 *in vitro* transcribed sgRNAs targeting the sequence ccg​TGG​GAG​GGA​AAA​CAA​TGC​TG for *sap130b* and cct​GCT​CCT​CTT​CAG​CCA​TAC​AG for *sin3ab*, where lower case letters represent the protospacer motif sequence. sgRNA was incubated at room temperature with Cas9 protein (NEB, Cat# M0646T). AB* embryos were injected at the one-cell stage with the sgRNA and Cas9 cocktail in a 1 nL volume at 25 pg sgRNA/nL. RFLP was performed to determine protected mutated bands present 24hrs after injection to determine gRNA efficiency and injected embryos were raised to adults outcrossed to AB*. DNA mutations in *sap130b* and *sin3ab* were verified by PCR TOPO-TA cloning (ThermoFisher, #K4575J10) from adult heterozygous animals and Sanger sequenced. gRNA sequence information Supplementary Table S1.

### Imaging

A Leica M205 FA stereomicroscope was used to take images of the hearts from *Tg(myl7:EGFP)* WT and mutant embryos at 36 and 48hpf. For imaging the *Tg(myl7:memGFP)* OFT, a Nikon A1 inverted confocal microscope was used at 72hpf. *Tg(myl7:memGFP)* embryos were anesthetized in 7x MS-222/10 mM BDM (2,3-butanedione monoxime) and mounted in low melting agarose on MaTek glass bottom petri dish (MaTek, Part No: P35G-1.5–14-C) and imaged at a 40x water immersion. For counting cardiomyocytes at 72hpf, *Tg (myl7:memGFP)* and *MZsap130a;Tg(myl7:EGFP)* were injected with 50 pg of *H2b-mCherry* mRNA at the 1-2 cell stage. Injected embryos were incubated at 28°C and mounted on an inverted confocal microscope at 72hpf on a Nikon A1 microscope. Ventricular cardiomyocytes were designated as positive for both *mCherry* nuclei and membrane GFP expression using Fiji ImageJ and the orthogonal views tool.

### ConSurf and R generated phylogenetic trees and protein diagram

ConSurf (https://consurf.tau.ac.il/consurf_index.php) was used to align multiple Sap130 protein sequences across many species (Berezin et al., 2004). The *sap130a* amino acid sequence from zebrafish was input to ConSurf and the output was collected and plotted in R, with ggtree, ggplot2 and phytools (Revell and Graham Reynolds, 2012; Wickham, 2016; Yu et al., 2018; Yu, 2020). A multiple sequence alignment (MSA) was performed on Sap130 protein sequences from UniProt and distance calculations to plot simple phylogeny trees using R CRAN packages seqinr, msa, Biostrings, ggtree, ggplot2 (Charif et al., 2005; Bodenhofer et al., 2015; Lifschitz et al., 2022). For plotting the protein sequences and conserved domains reported by UniProt, the R packages ggplot and drawProteins were used (Brennan, 2018).

### *In situ* probe synthesis, whole mount *in situ* hybridization

RNA probe generation and whole mount *in situ* hybridization for *nkx2.5*, *ltbp3*, *myh7* and *myh6* was performed as previously described with DIG RNA labeling kit (Millipore Sigma cat# 11175025910) (Znosko et al., 2010).

### RNAseq sample preparation and data analysis

Total RNA was extracted from whole embryos or isolated hearts (36hpf and 48hpf, respectively) using Trizol (Invitrogen) and was purified with the RNeasy Micro Kit (Qiagen#74004). A minimum 50 embryos or 180 hearts were pooled together for each condition. The RNA-seq used was 0.5–1 μg RNA for each condition and was sent to the Genomics Research Core at the University of Pittsburgh. The raw sequence reads were processed and mapped to the Zebrafish Reference Genome GRCz11 using CLC Genomics Workbench 20 RNAseq analysis tool. A count matrix was exported and the bioinformatic analysis was carried out in R (R Core Team, 2021) using the edgeR package for 36hpf whole embryo and 48hpf heart tissue data. Results for DEGs in Supplementary Tables, S2–S6 (Robinson et al., 2010). To identify cardiac changes with whole embryo resolution we defined DEGs as those with an FDR ≤ 0.05 and log2FC > ±0.4. After determining differentially expressed genes they were entered into DAVID (https://david.ncifcrf.gov/summary.jsp) for functional annotation clustering. Results for DAVID clustering in Supplementary Tables, S2–S6 (Sherman et al., 2022).

### Lineage tracing

Lineage tracing of cardiac progenitors at 24hpf was performed on *Tg(nkx2.5:kaede)* and *Tg(nkx2.5:kaede);sap130a* ^*pt32a/pt32a*^ embryos and was described by Guner-Ataman et al. (Guner-Ataman et al., 2013). Using the Zeiss Imager M2 confocal microscope at 40x, the ROI (Region of Interest) was selected to photoconvert the peristaltic heart tube at 24hpf. Embryos were mounted in low melting temperature agarose droplets on 35 mm dishes. The embryos were then freed from the agarose and raised in darkness until 48hpf, when the looped heart was imaged at 40x.

### Cardiac functional analysis

To measure cardiac function in embryonic zebrafish, we used a custom-built light sheet microscope which followed a design based on the openSPIM platform (Pitrone et al., 2013; Girstmair et al., 2016). This ‘T’ design illuminates the sample bilaterally and uses a four-channel laser launch for maximum versatility. *Tg(myl7:EGFP)* and *Tg(myl7:EGFP);sap130a* ^*m/m*^ embryos at 48hpf embryos were placed into E3 and Tricaine (307 nmol concentration) to anesthetize them before mounting for imaging. Low melting point agarose was heated and cooled to 42°C. 100 µL agarose placed onto a dish and after 45 s of cooling, 48hpf embryo was added to the agarose and drawn into a custom cut 1 mL straight-barreled syringe. The agarose is allowed to solidify, and the syringe is placed into a sample manipulator capable of 3D movement + rotation (Picard Technologies, Inc.). The agarose-embedded embryos were extruded from the syringe and positioned in a lateral view, with anterior to the left and posterior to the right, before recording 100 frames at 50–75 frames per second using a Prime 95B sCMOS camera (Photometrics, Inc.). Fiji ImageJ software was used to identify end-diastole and end-systole frames to calculate ventricle area, length (distance between ventricular apex and out-flow tract opening), and diameter for each embryo (distance between the walls of the chamber, taken from the middle of length measurement). These data were used to estimate chamber volumes and calculate end-diastole and systole volumes, ejection fraction (%), fractional shortening (µm), Total stroke volume, cardiac output, and heart rate as an average of all cycles captured for each fish. The volumes calculated are under the assumption of a prolate sphere shape (pi/6). The equations used are as follows (Yalcin et al., 2017);

$$Ejection\;Fraction\;\left(\%\right)=\frac{SV}{EDV}*100$$

$$End\_Diastole\;\&\;End\_Systole\;Volumes\;\left(EDV\;\&\;ESV\right)=\frac{\pi }{6}*Length*{Diameter}^{2}$$

$$Stoke\;Volume\;\left(SV\right)=End\_Diastole\;Volume-End\_Systole\;Volume$$

$$Fractional\;Shortening=\frac{Diastole\;diameter-Systole\;diameter}{Diastole\;diameter}$$

$$Fractional\;Area\;Change=\frac{End\_Diastole\;Area-End\_Systole\;Area}{End\_Diastole\;Area}*100$$

$$Heart\;Rate=\frac{\#\;of\;Cycles}{Aquisition\;time\;\left(s\right)}$$

These were implemented using R scripting and RStudio to automate the calculations, and then data were plotted using Graphpad PRISM 9.3. Each data point represents an average of 3 or more contraction cycles per fish (Yalcin et al., 2017).

### Adult heart measurements

At 48hpf *MZsap130a* mutant embryos were scored for ventricle size and raised in separate tanks. *MZsap130a* mutants and aged matched AB* controls were measured for length and weight before hearts were extracted for DIC imaging at 4-6mpf. Fiji-ImageJ was used to measure the ventricle surface area and bulbus arteriosus surface area. These data were plotted using Graphpad Prism 9.3.

### Statistics

For analysis of RNA-seq data we used the edgeR package, utilizing a quasi-likelihood negative binomial generalized log-linear model to our count data comparing AB* control to *MZsap130a* or *MZsap130b* mutant embryos at 36hpf. For heart tissue RNA-seq, edgeR’s likelihood ratio test was used to interpret up or downregulation of genes. For all other statistical analysis, significance was calculated using two-tailed, unpaired Student’s t-test, one-way ANOVA or Fisher’s exact text using GraphPad Prism version 9.3.

## Results

### *sap130b* is not required for heart development

**FIGURE 1:**
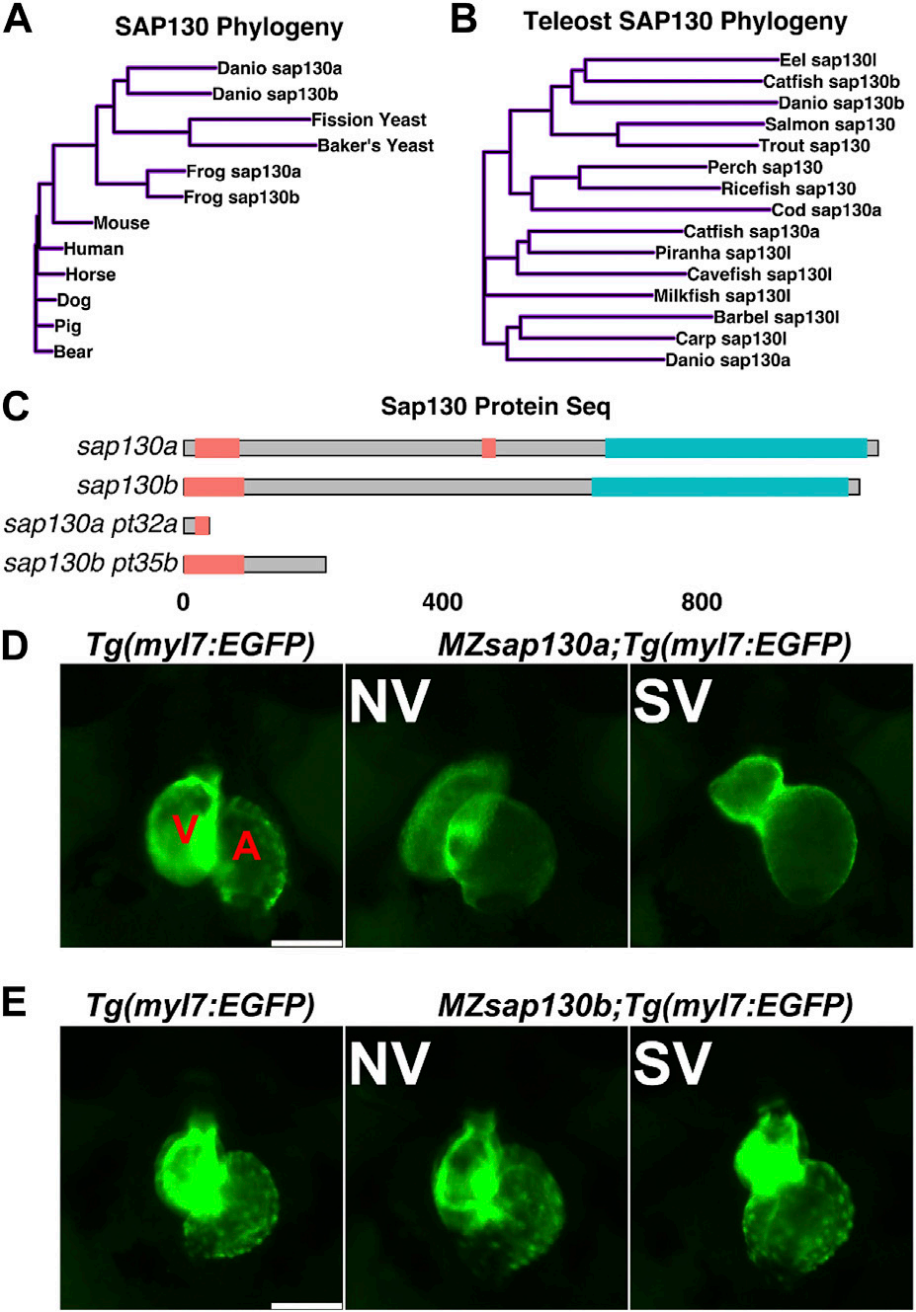
*sap130a* and *sap130b* have non-overlapping functions in the zebrafish heart **(A, B)** A simple distance matrix phylogeny tree of Sap130a and Sap130b in broad or teleost specific contexts **(C)** Schematic of Sap130a and Sap130b protein sequences from the UniProt database highlighting the conserved regions and predicted mutant proteins. Unorganized sequence in pink, C-terminal conserved domain in blue, which contains the binding domain for SIN3A and HDAC1 **(D, E)** Representative images of *Tg*(*myl7:EGFP*), *MZsap130a;Tg(myl7:EGFP)* and *MZsap130b;Tg(myl7:EGFP)* mutant hearts at 48hpf. V and A are ventricle and atria, respectively. Scale bar 100 μm.

**FIGURE 2:**
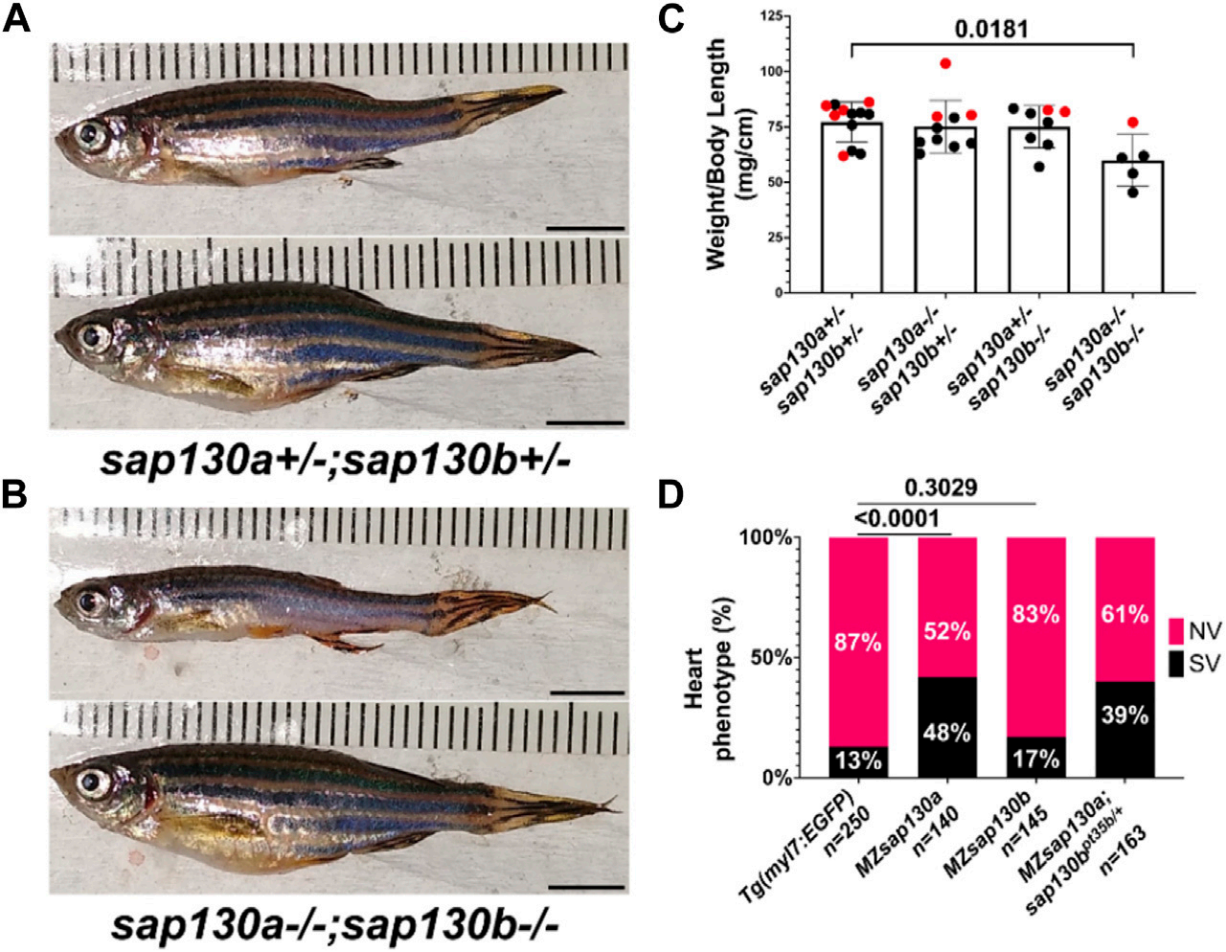
*MZsap130a;sap130b* ^*pt35b/pt35b*^ mutants are not healthy **(A)** *sap130a*;*sap130b* double heterozygous adults, male (top) and female (bottom). **(B)** *sap130a*;*sap130b* double homozygous adults, male (top) and female (bottom). **(C)** Graph quantifying weight to length ratio for adults from a *sap130a*;*sap130b* double heterozygous in-cross, pvals are for one-way ANOVA, error bars are standard error mean (SEM). Red points represent females and males in black. **(D)** Graph quantifying the heart phenotype proportions for *Tg*(*myl7:EGFP*), *MZsap130a;Tg(myl7:EGFP)*, *MZsap130b;Tg*(*myl7:EGFP*), and *MZsap130a;sap130b* ^*pt35b/+*^ *;Tg*(*myl7:EGFP*)**,** pvals are for fisher’s exact test. Scale bar 5 mm.

Zebrafish were part of the teleost-specific genome duplication event 350 million years ago (Alsop and Vijayan, 2009), resulting in two *sap130* genes, *sap130a* and *sap130b*. Defining the SAP130 protein domains based on homology with other model organisms will provide insight into the potential conserved functional domains. In mammals, both SIN3A and HDAC1 proteins were shown to interact with SAP130 at the C-terminus between amino acids 836–1,047, suggesting that SAP130 may act as a stabilizing scaffold between these proteins (Fleischer et al., 2003). Determining protein sequence similarities can predict functional structures across species and offer insight into the potential for functional redundancy between Sap130a and Sap130b. ConSurf was used for a multispecies comparison of 145 unique SAP130 protein sequences to determine their similarity and conserved domains (Berezin et al., 2004). In general, Sap130a and Sap130b are dissimilar, but they both contained conserved N- and C-terminus domains represented by repetitive predicted structural and functional residues (Supplementary Figure S1). Comparing SAP130 proteins to a small group of common species Sap130a and Sap130b are most like one another, suggesting they could serve similar functions (Figure 1A). Narrowing the comparison to a smaller set of protein sequences among other teleost, Sap130a and Sap130b are distinct suggesting in teleost these genes could have evolved distinct functions (Figure 1B). However, given that the C- terminal domains are most conserved, Sap130a and Sap130b can potentially compensate for one another in zebrafish (Figure 1C). *MZsap130a* mutants develop SVs in 36% of the population by 72hpf (Liu et al., 2017). The incomplete penetrance of the SV phenotype was hypothesized to be the result of *sap130b* compensating for the loss of *sap130a*. To address this, we generated a mutation in *sap130b* using CRISPR/Cas9 technology. This produced an allele (7bp del, 1bp sub (G>C))*sap130b* ^*pt35b/pt35b*^ that introduced a premature stop codon in exon 6 of *sap130b* disrupting the N-terminus and eliminating the C-terminal region (Figure 1C, Supplementary Tables S1). Using the *Tg(myl7:EGFP)* line, which labels the heart with green fluorescent protein, we found that 48% of the *MZsap130a;Tg(myl7:EGFP)* mutant embryos had the SV heart phenotype at 48hpf (Figure 1D). In contrast, only 17% of the *MZsap130b;Tg(myl7:EGFP)* mutant embryos had SVs by 48hpf (Figure 1E). We generated double mutants to further explore if *sap130a* and *sap130b* have any redundant functions (Figures 2A, B). The offspring produced the expected number of double mutants (7/120 (5.8%)) from the expected (1/16 (6.25%)) from a double heterozygous in-cross. However, the adult double *sap130a/b* mutants are much smaller than their double heterozygous siblings and failed to produce offspring when bred (Figure 2C). *MZsap130a;sap130b* ^*pt35b/+*^ mutant in-crosses, resulted in 39% of the embryos with SVs at 48hpf, which is in the same range as *MZsap130a* mutants indicating the zygotic loss of *sap130b* did not contribute to increased cardiac defects (Figure 2D). These observations suggest *sap130b* is not required for zebrafish cardiogenesis.

**FIGURE 3:**
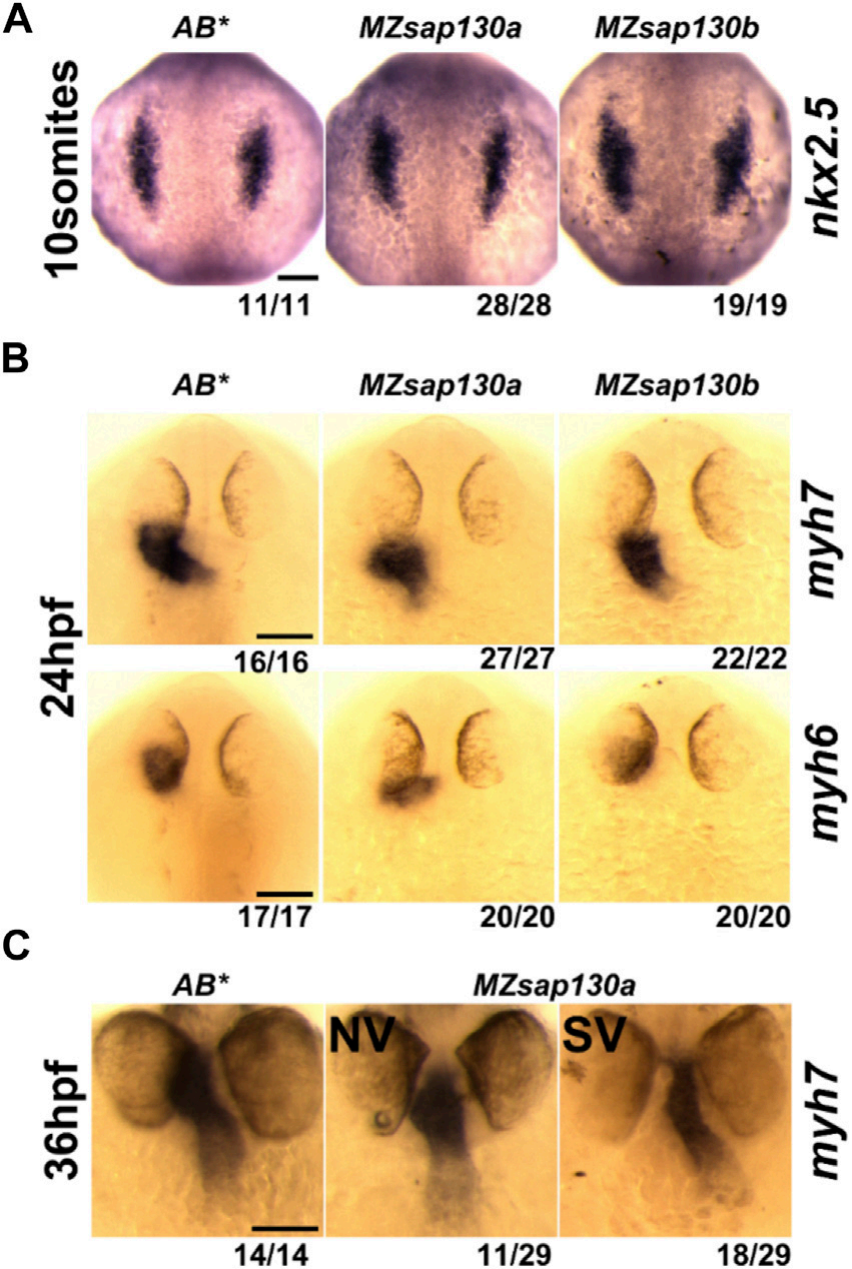
Cardiac gene expression in *MZsap130a* and *MZsap130b* **(A)** WISH of *nkx2.5* at 10 somite stage for AB*, *MZsap130a* and *MZsap130b*. **(B)** WISH of *myh6* and *myh7* at 24hpf for AB*, *MZsap130a* and *MZsap130b*. **(C)** WISH of *myh7* at 36hpf in AB* and *MZsap130a*. Scale bar 100 μm.

**FIGURE 4:**
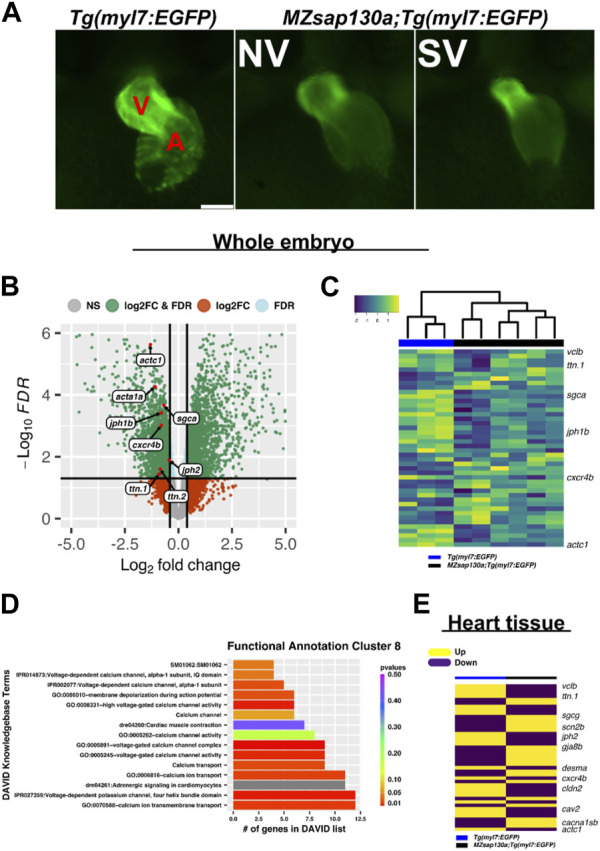
RNAseq reveals cardiac contraction and conduction is altered in *MZsap130a* **(A)** Representative images of *Tg*(*myl7:EGFP*) and *MZsap130a;Tg(myl7:EGFP)* embryos collected for whole embryo RNAseq at 36hpf. **(B)** Volcano plot of 36hpf whole embryo RNAseq data. **(C)** Heatmap of sarcomere and conduction genes (Supplementary Figure S6) at 36hpf from whole embryos. **(D)** DAVID functional annotation cluster 8 from downregulated genes in *MZsap130a*, showing DAVID calculated *p*-values. **(E)** Heatmap of 48hpf heart tissue RNAseq data for the same genes found in panel C. V and A are ventricle and atria, respectively. Scale bar 100 μm.

*Sap130a* AUG start codon antisense-morpholino (MO) studies suggested the SVs arise from decreased ventricular CMs (Liu et al., 2017), but where or when CMs are lost was not explored. To determine if the SVs are due to decreased cardiac progenitors, we performed Whole Mount *In Situ* Hybridization (WISH) at 10 somite stage with *nkx2.5*, an early cardiac progenitor marker. We discovered no differences between *MZsap130a* and controls (Figure 3A). This suggests that the early cardiac progenitors were present in the *MZsap130a* embryos. To profile a later stage of the First Heart Field (FHF) and the chambers of the heart we performed WISH at 24hpf with myosin heavy chain 7 (*myh7*, ventricle) and myosin heavy chain 6 (*myh6*, atria). No difference between WT and mutant embryos were observed, suggesting the FHF is intact (Figure 3B). At 36hpf and 48hpf the atrial chamber showed no change, but the ventricle was smaller (Figure 3C, Supplementary Figure S2). This phenotype was observed again when imaging the *MZsap130a;Tg(myl7:EGFP)* at 36hpf (Figure 4A). Many studies have detailed the second heart field accretion between 24 and 48hpf in zebrafish (Grimes et al., 2008; de Pater et al., 2009; Hami et al., 2011; Lazic and Scott, 2011). These SHF cells trail behind the heart tube and add to the ventricle continuously. There is speculation as to how many SHF cells are ventricular CMs, between 30%–40% of the total ventricular CMs by 48hpf has been proposed (Felker et al., 2018). The SV heart phenotype arising at 36hpf and the lack of changes seen in FHF markers suggest the SHF might be an influenced cell population where CMs are lost in *MZsap130a* mutants.

### RNAseq reveals *sap130a* is involved in regulating cardiac sarcomere and conduction genes

Variants in genes encoding sarcomere proteins have been linked to CHDs (Jones et al., 1996; Morano et al., 2000; Ching et al., 2005; Zhu et al., 2006; Monserrat et al., 2007; Granados-Riveron et al., 2010; Postma et al., 2011; Fahed et al., 2013; van Engelen et al., 2013). The Sin3A complex has been shown to regulate sarcomere specific genes like titins, troponins, and actins important for cardiac contraction (van Oevelen et al., 2010). Since SAP130 has been shown to be part of the SIN3A complex, we reasoned that the phenotype may be caused by altered regulation of cardiac gene expression during development. A whole embryo RNA sequencing (RNAseq) experiment, separating the SV and “normal” (NV) siblings in the *MZsap130a* mutants, was performed at 36hpf. We first performed our analysis looking for differences in the wildtype, compared to NV and SV separately finding 2,826 differentially expressed genes (DEGs) in common, with 812 unique DEGs for NV and 1979 for SV. Functional annotation of these gene groups revealed that NV and SV embryos are similar when compared to the wildtype transcriptome (Supplementary Table S2). Comparing the controls to all *MZsap130a* samples (both NV and SV), we observed 5,002 DEGs that included many cardiac specific transcripts. Among the DEGs we found sarcomere and cardiac conduction genes were dysregulated, suggesting CM biology has changed in the *MZsap130a* embryos (Figures 4B, C, Supplementary Tables S3, S4). To identify potential pathways involved in heart function and development, we used the Database for Annotation, Visualization, and Integrated Discovery (DAVID) functional annotation of downregulated genes. This showed enrichment for cardiac contraction and adrenergic signaling in CMs, further suggesting a role for *sap130a* in CM function (Figure 4D, Supplementary Table S6). To confirm cardiac specific changes in these same transcripts, *MZsap130a* mutant hearts and controls were harvested at 48hpf and the transcriptome was profiled, showing similar cardiac gene expression changes (Figure 4E, Supplementary Table S5). *MZsap130b* whole embryo transcriptome was also profiled at 36hpf and less gene expression changes (617 DEGs) were noted (Supplementary Figure S3, Supplementary Table S6). Moreover, the sarcomere gene expression changes seen in *MZsap130a* was not detected in the *MZsap130b* transcriptome. DAVID functional annotation of the 278 DEGs common between *MZsap130a* and *MZsap130b* mutants, belonged to heme binding and biosynthesis, oxygen binding, and iron binding KEGG pathways, suggesting involvement in hematopoiesis (Supplementary Table S6). The expression profile for these hematopoietic related genes was opposite in *MZsap130a* and *MZsap130b*, suggesting distinct functions during hematopoiesis (Supplementary Figure S3). These data suggest that *sap130a* and *sap130b* could be involved in hematopoiesis that correlates with *sin3aa*/*ab* gene knockdown studies showing strong hematopoietic defects (Huang et al., 2013).

Whole embryo and heart tissue *MZsap130a* RNA-seq data revealed sarcomere genes such as actins and myosins were dysregulated, indicating that sarcomere dysfunction could be part for the *MZsap130a* mutant phenotype. These data also showed downregulation of CM conduction genes such as *cxcr4b* and *gja3*, resulting in changes in cardiogenesis (Severs et al., 2004; Stankunas et al., 2008; Chi et al., 2010; Itou et al., 2012; Mortensen et al., 2017; Jiang et al., 2019). *dococ* ^*s226*^ (*gja3*) mutants report having changes in cardiac conduction that lead to CM morphological changes in the ventricle (Chi et al., 2010). Rat studies have shown *Cxcr4* involvement in cardiac conduction (Pyo et al., 2006). While *MZcxcr4b* mutants are reported to have abnormal organ morphogenesis, including heart looping defects (Jiang et al., 2019). Changes were found in calcium channel (*cacna1sb*, *cacng7a*, *cacnb1*, *cacna1bb*) and sodium channel (*scn4aa*/*ab*, *scn2b*) genes, known to be important to CM biology (Haverinen et al., 2018; Papa et al., 2022; Shah et al., 2022). Furthermore, transcriptome analysis revealed that *MZsap130a* mutants showed dysregulation of a wide range of genes critical for cardiac maturation and function. These include genes associated with fatty acid metabolism (*ppt2*), glycogen metabolism (*ugp2a*, *phka2*), and mitochondria (*slc25a44a*, *slc25a42*, *mtrf1*, *mrpl58*) found downregulated in *MZsap130a* mutants in whole embryos at 36hpf and specifically in the heart at 48hpf (Supplementary Figure S3). Deficits in mitochondrial function have been shown in HLHS patients and other HLHS models including *Sap130* mouse mutants and *rbfox* mutant zebrafish (Liu et al., 2017; Huang et al., 2022). Collectively, *MZsap130a* mutants show changes in sarcomere, conduction and metabolism associated genes, all integral parts of CM maturation and function.

### Sap130a regulates cardiac function

**FIGURE 5:**
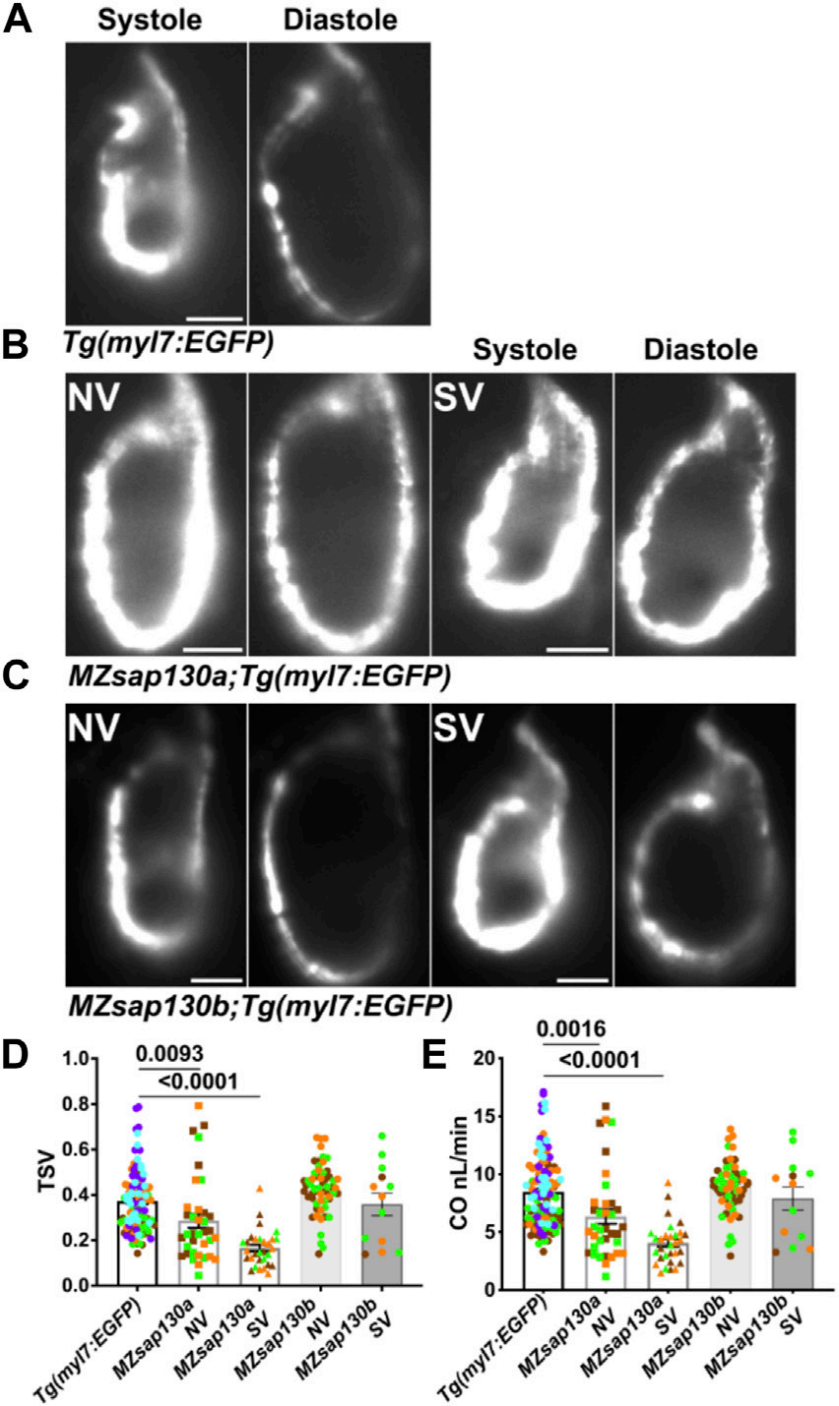
*MZsap130a* show cardiac functional deficits **(A–C)** Shows systole and diastole frames from recordings of live ventricular contractions in *Tg*(*myl7:EGFP*), *MZsap130a;Tg(myl7:EGFP)* and *MZsap130b;Tg*(*myl7:EGFP*) at 48hpf. **(D, E)** Quantified cardiac parameters total stroke volume (TSV) and cardiac output (CO), pvals from one-way ANOVA, error bars are SEM. Each point represents individual ventricle and color coded for 3+ experiments. For *Tg(myl7:EGFP)*, *n* = 115; *MZsap130a* NV, *n* = 36; *MZsap130a* SV, *n* = 30; *MZsap130b* NV, *n* = 57; *MZsap130b* SV, *n* = 13. Scale bar 20 μm.

Global loss of *sap130a* showed downregulation of sarcomere genes such as *actc1*, *ttn.1*, and *ttn.2* (Figures 4B–E **)**. This suggested that cardiac function could be diminished in *MZsap130a* mutants. The DAVID functional annotation tool revealed enrichment for cardiac muscle contraction genes that were decreased in the *MZsap130a* mutant embryos (Figure 4D). To determine ventricle chamber function in mutants, confocal light sheet microscopy was used to record live cardiac contractions at 48hpf. These recordings provided us with multiple frames of diastole and systole for chamber volume estimation (Figures 5A–C, and Supplementary Movie S1–S5). Volume estimations were used to calculate the cardiac parameters Total Stroke Volume (TSV), and Cardiac Output (CO) (Yalcin et al., 2017). The light sheet data revealed that all *MZsap130a* mutants had deficits in CO, TSV, fractional shortening, and ejection fraction (Figures 5D, E, and Supplementary Figure S5). The *MZsap130b* mutant hearts revealed no significant difference from WT function, both in TSV and CO, but showed an increase in End-systolic volume which could explain the increase in CO through increased contraction force (Figures 5D, E and Supplementary Figure S5). The heart tissue RNA-seq identified cardiac contraction genes *myh7*, *actc1*, *ttn.1*, *ttn.2*, *scn4ab*, and *cacna1sb* were dysregulated in *MZsap130a* mutants, supporting the contraction deficits measured at 48hpf (Supplementary Figure S6). These data show that *sap130a* has a role in zebrafish cardiac sarcomere regulation.

### *MZsap130a* mutants have longer outflow tract

**FIGURE 6:**
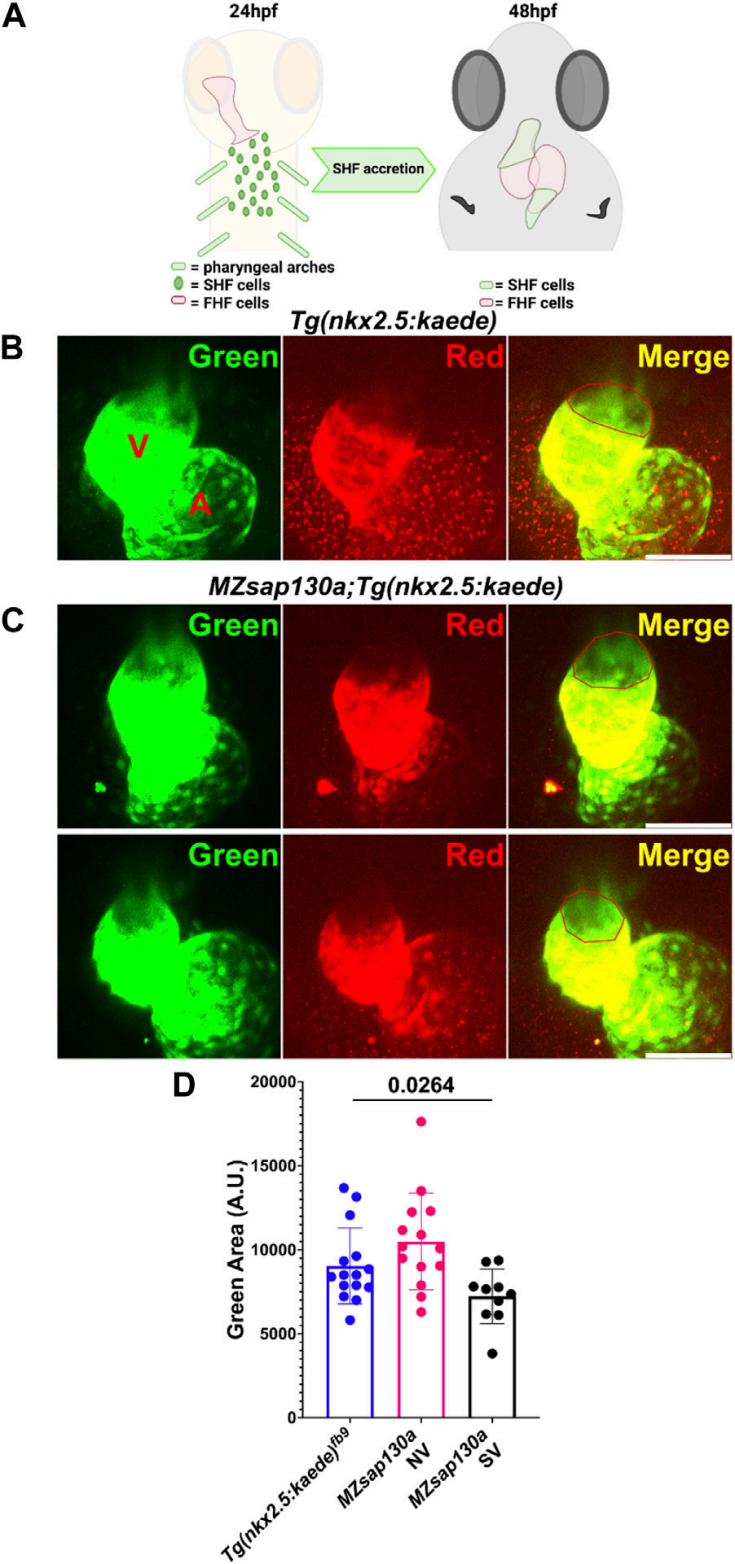
Lineage tracing reveal changes to SHF in *MZsap130a* at 48hpf **(A)** Diagram showing how the FHF heart tube at 24hpf was photoconverted to red, leaving SHF progenitors unlabeled in green and imaging at 48hpf. **(B, C)** Confocal imagines of *Tg(nkx2.5:kedge)* and *MZsap130a;Tg(nkx2.5:kedge)* at 48hpf with the heart tube being photoconverted at 24hpf, the red outlined region represents area measurements collected. **(D)** Quantified SHF (green area) accreted by 48hpf, pval is from a one ANOVA, error bars are SEM. Each point represents a single embryo, *Tg(nkx2.5:kedge)*, *n* = 15; *MZsap130a;Tg(nkx2.5:kedge)* NV, *n* = 14; *MZsap130a;Tg(nkx2.5:kedge)* SV, *n* = 10. V and A are ventricle and atria, respectively. Scale bar 100 μm.

**FIGURE 7:**
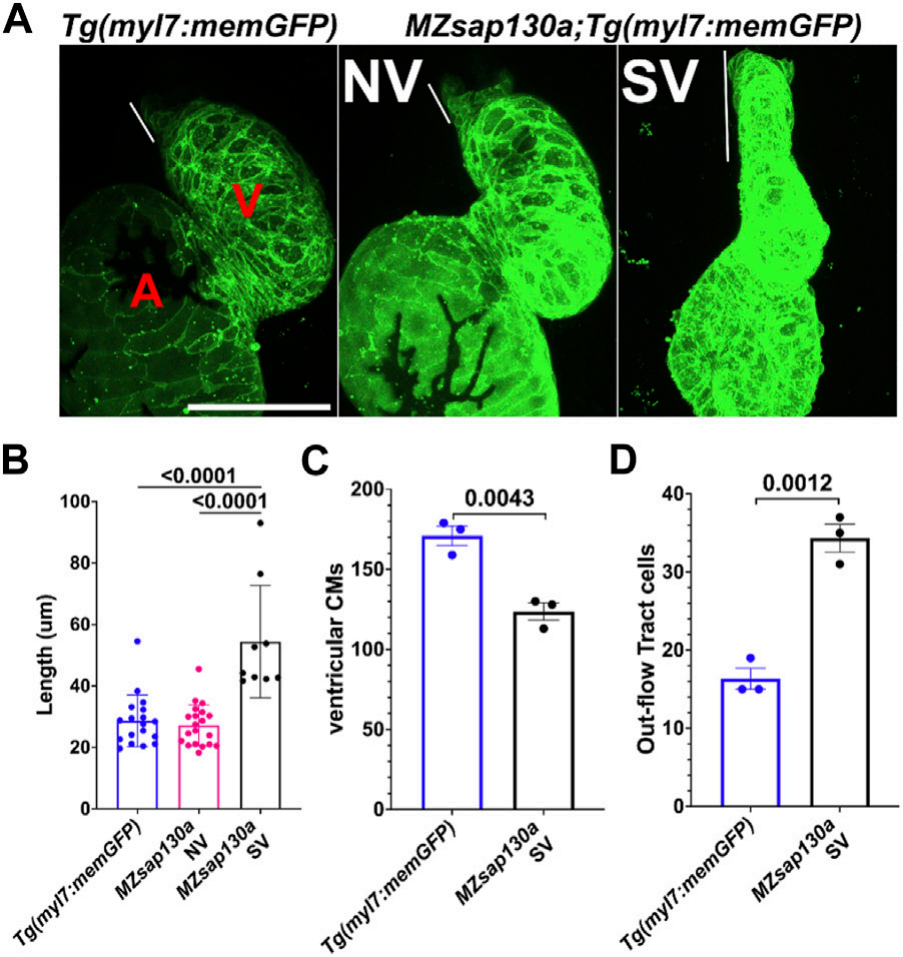
*Tg(myl7:memGFP)* reveals longer OFT by 72hpf in *MZsap130a* **(A)** Quantified OFT lengths at 72hpf, pval is from a one ANOVA, SEM error bars. Each point represents a single embryo, *Tg(myl7:memGFP)*, *n* = 18; *MZsap130a;Tg(myl7:memGFP)* NV, *n* = 20; *MZsap130a;Tg(myl7:memGFP)* SV, *n* = 9 **(B)** Representative images of *Tg(myl7:memGFP)* and *MZsap130a;Tg(myl7:memGFP)*, white lines demarcate OFT length, pvals from one way ANOVA, SEM error bars. **(C)** *Tg(myl7:memGFP)+;H2b:mCherry +* ventricular CM counts for three WT or *MZsap130a* SV heart, pval is from a t-test, SEM error bars **(D)** *Tg(myl7:memGFP)+;H2b:mCherry +* out-flow tract cell counts for three WT or *MZsap130a* SV heart, pval is from a t-test, SEM error bars. V and A are ventricle and atria, respectively. Scale bar 100 μm.

**FIGURE 8:**
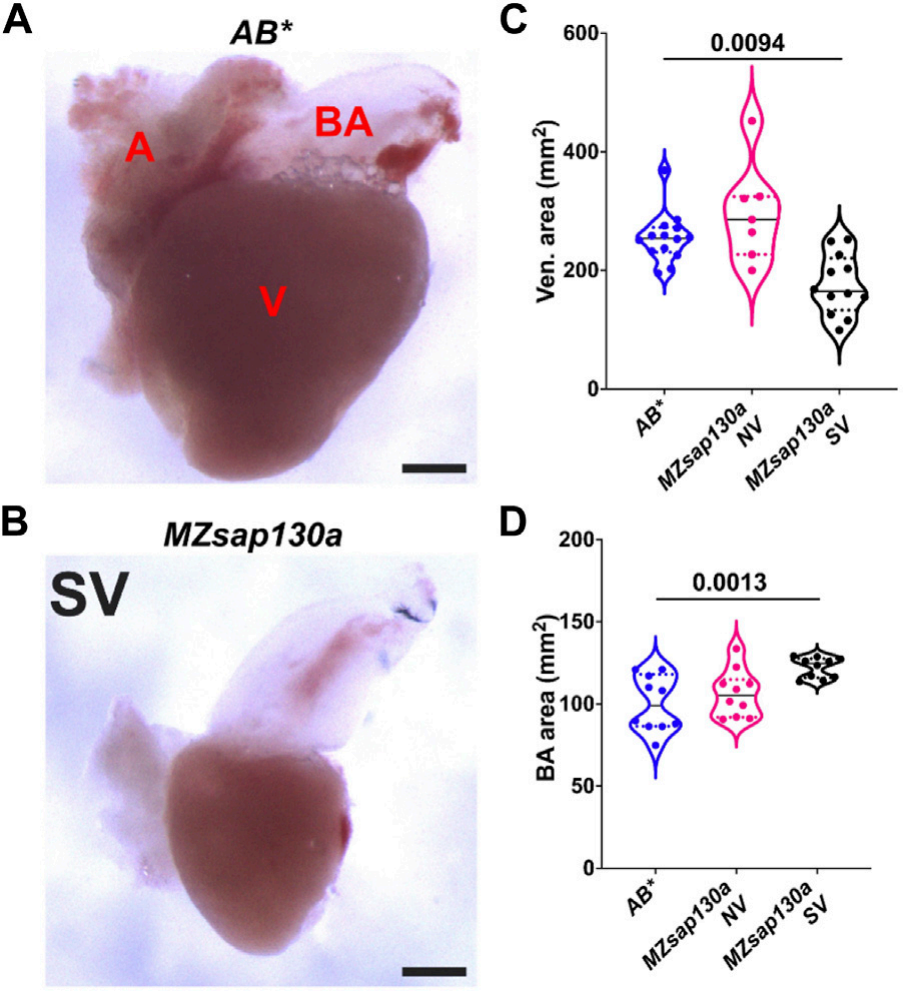
Adult *MZsap130a* hearts have large bulbus arteriosus **(A, B)** AB* and *MZsap130a* adult SV hearts extracted at approximately 4–6 months post fertilization. **(C)** Quantification of ventricle area of unfixed hearts, pval is from a one ANOVA solid black bars are mean, dotted lines represent up and lower 25th percentiles. Each point represents a single heart, AB*, n = 14; *MZsap130a* NV, *n* = 7; *MZsap130a* SV, *n* = 12. **(D)** Quantification of BA area. of unfixed hearts, pval is from a one ANOVA solid black bars are mean, dotted lines represent up and lower 25th percentiles. Each point represents a single heart, *n* = 10 for all groups. V, A, and BA are ventricle, atria, and bulbus arteriosus respectively, Scale bar 200 μm.

The earliest observation of smaller ventricles in *MZsap130a* mutants was at 36hpf, a stage when SHF cells are migrating into the ventricle. Extensive studies have reported the contribution of SHF cells to the ventricle during this time (Grimes et al., 2008; de Pater et al., 2009; Lazic and Scott, 2011; Knight and Yelon, 2016; Felker et al., 2018; Song et al., 2019). RNA-seq data showed that SHF progenitor markers *ltbp3*, *mef2cb* and *isl1, isl2a/b* were decreased (Supplementary Figure S7). These genes are known to label SHF progenitors at the arterial and venous poles. WISH at 30hpf revealed a decrease in *ltbp3* expression in *MZsap130a* mutants (Supplementary Figure S8). Together these data suggest that the SHF in the *MZsap130a* mutants was affected such that insufficient CMs contribute to the ventricle by 48hpf. To determine if this occurs, we performed lineage tracing experiments using *Tg(nkx2.5:kaede)* embryos (Guner-Ataman et al., 2013). In this transgenic line, the FHF cells can be permanently labeled at 24hpf, photo-converting only the heart tube. Next, we imaged at 48hpf to determine the addition of green cells to the ventricle (Figure 6A, and Supplementary Figure S9. Lineage tracing the SHF with *MZsap130a;Tg(nkx2.5:kaede)* embryos revealed that the SVs acquire less SHF (green area) compared to the wildtype and *MZsap130a* mutant siblings that develop normal ventricles (Figures 6B–D). Moreover, the OFTs in the *MZsap130a* mutants were longer at 48hpf in some embryos with SVs (Supplementary Figure S10). The longer OFTs were much more pronounced at 72hpf, and every SV heart had a longer OFT (Figures 7A, B, and Supplementary Figure S10). To count the CMs in the ventricle and OFT of the *MZsap130a* mutants, we injected *Tg(myl7:memGFP)* and *MZsap130a;Tg(myl7:memGFP)* embryos with *H2b:mCherry* mRNA. We detected an increase in OFT cells that was concomitant with a decrease in ventricular CMs (Figures 7C, D and Supplementary Figure S11). This suggested that the lost ventricular CMs contributed to OFT cells instead and was further evidenced at adult stages. *MZsap130a;Tg(myl7:EGFP)* embryos were scored at 48hpf for ventricle size and reared separately into adulthood. Images of heart revealed a larger bulbus arteriosus (BA) area, the adult structure derived from the OFT, and decreased ventricular area (Figure 8). The observations in the *MZsap130a* adults from small ventricle embryos correlates with the notion that *sap130a* is involved in SHF cell fate decisions between ventricular CMs and OFT cells.

### *sap130a* genetically interacts with *hdac1* during SHF accretion

**FIGURE 9:**
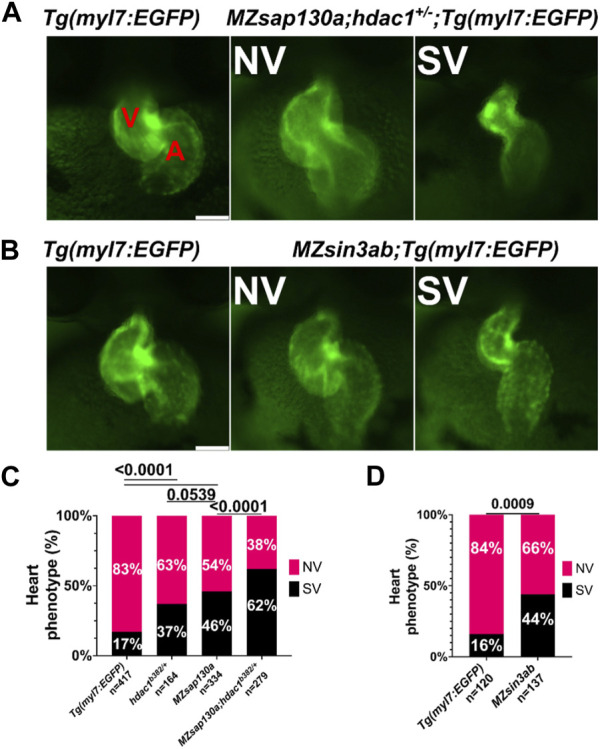
*sap130a* shows an association with *hdac1* and *MZsin3ab* mutants have SVs **(A)** Representative image of *Tg(myl7:EGFP)* and *MZsap130a;hdac1* ^*b382/+*^ *;Tg*(*myl7:EGFP*) NV and SVs at 48hpf. **(B)** Representative image of *Tg(myl7:EGFP)* and *MZsin3ab*;*Tg(myl7:EGFP)* NV and SVs at 48hpf. **(C)** Quantification of heart phenotype proportions in *Tg(myl7:EGFP)*, *MZsap130a;Tg(myl7:EGFP)*, *hdac1* ^*b382/+*^ *;Tg*(*myl7:EGFP*) and *MZsap130a;hdac1* ^*b382/+*^ *;Tg*(*myl7:EGFP*). The *p*-values are fisher’s exact test. **(D)** Quantification of heart phenotype proportions in *Tg(myl7:EGFP)* and *MZsin3ab;Tg(myl7:EGFP).* The *p*-values are fisher’s exact test. V and A are ventricle and atria, respectively. Scale bar 100 μm.

Zebrafish *hdac1* is required for ventricle formation (Song et al., 2019; Buhler et al., 2021). We explored the potential interaction of Sap130a and Hdac1 by analyzing heart development in *MZsap130a;hdac1* ^*+/b382*^ embryos. While *hdac1* homozygous mutants develop cardiac defects, heterozygous mutants are viable and show a similar proportion of SVs like in the *MZsap130a* mutants. An increase in SV phenotype was noted in *MZsap130a;hdac1* ^*+/b382*^ suggesting *MZsap130a* mutants are sensitized to *hdac1* gene dosage (Figures 9A, C). These data revealed an association between *hdac1* heterozygous status and ventricle size and this increase in a *MZsap130a* background (Figure 9C). This suggests that *sap130a* and *hdac1* genetically interact in zebrafish and function in the same complex like in mammals.

Both *MZsap130a* whole embryo and heart specific RNA-seq datasets revealed sarcomere genes to be down and cell cycle genes to be upregulated, similar to SIN3A knock-out and knock-down studies (Supplementary Figure S12) (van Oevelen et al., 2008; van Oevelen et al., 2010; Dobi et al., 2014). For example, the cell cycle genes *vrk1*, *e2f7*, and *haus6* are increased in *MZsap130a* mutants, while we did not find evidence of expanded cardiac progenitors. These similarities in up and down DEGs point to the possibility that *sap130a* associates with *sin3aa* or *sin3ab* in zebrafish, similar to mammals. To further explore the importance of SIN3A in heart development, we generated *MZsin3ab* mutants using CRISPR/Cas9. The *sin3ab pt36a* allele generated disrupted amino acids 862–867. In *MZsin3ab* mutants an in complete penetrant SV phenotype was observed in 44% (Figures 9B, D). WISH data at 30hpf, revealed that *ltbp3* expression in *MZsin3ab* mutants was reduced, similar to the *MZsap130a* mutants in Supplementary Figure S8. It is not surprising that the penetrance of the phenotype in *MZsin3ab* was also incomplete since both *sin3aa* and *sin3b* could compensate for the disruption of *sin3ab*. These data suggest that *sin3ab* is involved in ventricular development in zebrafish, a phenotype that is reminiscent of *sap130a* and *hdac1* mutants.

## Discussion

In this study, we have revealed a role for *sap130a* in zebrafish cardiogenesis. We describe a null allele of *sap130a*, resulting in small ventricles through the delay and failure of SHF cells to migrate into the ventricle. Without *sap130a*, some of the SHF progenitors permanently become OFT cells. Transcriptome profiling of the *MZsap130a* embryos at 36hpf and hearts at 48hpf revealed that expression of sarcomere, conduction, and metabolism genes were dysregulated. This suggest that the CMs fail to terminally differentiate and properly function.

Our study reveals the consequence of disrupting members of the SIN3A complex, resulting in improper heart development. In the *MZsap130a* mutants, the main phenotype is a small ventricle leading to larger OFT and bulbus arteriosus in adulthood. Developmentally this arises from the failure of SHF progenitors to migrate into the growing ventricle. We come to this conclusion because the WISH data for *nkx2.5* and *myh7* showed no changes prior to the 24 hpf, indicating the FHF is intact. The phenotype arising at 36hpf is in line with observations showing the addition of SHF cells between 24 and 48hpf and with our lineage tracing experiments (Grimes et al., 2008; de Pater et al., 2009; Hami et al., 2011; Lazic and Scott, 2011; Felker et al., 2018). In the *Ohia* mouse mutant, the combination of *PCDHA9* and a *SAP130* mutations caused an HLHS etiology influencing the FHF structures. The prominent phenotype included a hypoplastic left ventricle and valve abnormalities in 11% of mouse embryos. In the zebrafish, the *sap130a* mutation is predicted to be a null mutant producing a hypoplastic ventricle in 48% of embryos. The difference seen between the mouse and zebrafish can be explained by the difference in the number of ventricle chambers, the specialized development of the mammalian OFT, and the changes seen during the evolution of this specialized pump from sea to land (Jensen et al., 2013). A recent study of *SAP130* pig CRISPR mutants show tricuspid dysplasia and atresia, highlighting the complex role of Sap130 in heart development across different species (Gabriel et al., 2021).

The catalytic unit of the SIN3A complex is comprised of class I HDACs, which deacetylate lysine residues to alter gene expression or protein function. The *hdac1* mutant, *cardiac really gone (crg)* exhibit decreased ventricular CMs by 36hpf (Song et al., 2019). This is also the timepoint *MZsap130a* mutants show decreased ventricular size. This suggests *sap130a* and *hdac1* could have overlapping functions during SHF development. Although SAP130 was shown to have HDAC-independent functions from *in vitro* studies (Fleischer et al., 2003), genetic studies suggest *sap130a* and *hdac1* interact for proper ventricular cardiomyocyte development. This supports a model in which Sap130a associates with the Sin3a/Hdac1 complex and/or an Hdac1-independent X-factor to regulate transcription (Supplementary Figure S13).

Taken together this study support the importance of Sap130a/Sin3a/Hdac complex in zebrafish cardiogenesis. These observations build upon previous studies in zebrafish *hdac1* and reiterates the importance of context specific components for the Sin3a complex during cardiogenesis.

## Data Availability

The datasets presented in this study can be found in online repositories. The names of the repository/repositories and accession number(s) can be found below: NCBI GEO under GSE228451.

## References

[B1] Adams G. E. Chandru A. Cowley S. M. (2018). Co-Repressor, co-activator and general transcription factor: the many faces of the Sin3 histone deacetylase (HDAC) complex. Biochem. J. 475 (24), 3921–3932. 10.1042/BCJ20170314 30552170 PMC6295471

[B2] Alfert A. Moreno N. Kerl K. (2019). The BAF complex in development and disease. Epigenetics Chromatin 12 (1), 19. 10.1186/s13072-019-0264-y 30898143 PMC6427853

[B3] Alsop D. Vijayan M. (2009). The zebrafish stress axis: molecular fallout from the teleost-specific genome duplication event. Gen. Comp. Endocrinol. 161 (1), 62–66. 10.1016/j.ygcen.2008.09.011 18930731

[B4] Auman H. J. Fernandes I. H. Berrios-Otero C. A. Colombo S. Yelon D. (2023). Zebrafish smarcc1a mutants reveal requirements for BAF chromatin remodeling complexes in distinguishing the atrioventricular canal from the cardiac chambers. Dev. Dyn. 10.1002/dvdy.595 PMC10589389 37083132

[B5] Barron D. J. Kilby M. D. Davies B. Wright J. G. Jones T. J. Brawn W. J. (2009). Hypoplastic left heart syndrome. Lancet 374 (9689), 551–564. 10.1016/S0140-6736(09)60563-8 19683641

[B6] Berezin C. Glaser F. Rosenberg J. Paz I. Pupko T. Fariselli P. (2004). ConSeq: the identification of functionally and structurally important residues in protein sequences. Bioinformatics 20 (8), 1322–1324. 10.1093/bioinformatics/bth070 14871869

[B7] Bisserier M. Mathiyalagan P. Zhang S. Elmastour F. Dorfmuller P. Humbert M. (2021). Regulation of the methylation and expression levels of the BMPR2 gene by SIN3a as a novel therapeutic mechanism in pulmonary arterial hypertension. Circulation 144 (1), 52–73. 10.1161/CIRCULATIONAHA.120.047978 34078089 PMC8293289

[B8] Bodenhofer U. Bonatesta E. Horejs-Kainrath C. Hochreiter S. (2015). msa: an R package for multiple sequence alignment. Bioinformatics 31 (24), 3997–3999. 10.1093/bioinformatics/btv494 26315911

[B9] Brennan P. (2018). drawProteins: a Bioconductor/R package for reproducible and programmatic generation of protein schematics. F1000Res 7, 1105. 10.12688/f1000research.14541.1 30210791 PMC6107989

[B10] Brown M. A. Sims R. J. Gottlieb P. D. Tucker P. W. (2006). Identification and characterization of Smyd2: A split SET/MYND domain-containing histone H3 lysine 36-specific methyltransferase that interacts with the Sin3 histone deacetylase complex. Mol. Cancer 5, 26. 10.1186/1476-4598-5-26 16805913 PMC1524980

[B11] Buhler A. Gahr B. M. Park D. D. Bertozzi A. Boos A. Dalvoy M. (2021). Histone deacetylase 1 controls cardiomyocyte proliferation during embryonic heart development and cardiac regeneration in zebrafish. PLoS Genet. 17 (11), e1009890. 10.1371/journal.pgen.1009890 34723970 PMC8584950

[B12] Carrozza M. J. Li B. Florens L. Suganuma T. Swanson S. K. Lee K. K. (2005). Histone H3 methylation by Set2 directs deacetylation of coding regions by Rpd3S to suppress spurious intragenic transcription. Cell 123 (4), 581–592. 10.1016/j.cell.2005.10.023 16286007

[B13] Charif D. Thioulouse J. Lobry J. R. Perriere G. (2005). Online synonymous codon usage analyses with the ade4 and seqinR packages. Bioinformatics 21 (4), 545–547. 10.1093/bioinformatics/bti037 15374859

[B14] Chen Q. Chen L. Jian J. Li J. Zhang X. (2022). The mechanism behind BAF60c in myocardial metabolism in rats with heart failure is through the PGC1α-PPARα-mTOR signaling pathway. Biochem. Cell Biol. 100 (2), 93–103. 10.1139/bcb-2019-0450 33245682

[B15] Chi N. C. Bussen M. Brand-Arzamendi K. Ding C. Olgin J. E. Shaw R. M. (2010). Cardiac conduction is required to preserve cardiac chamber morphology. Proc. Natl. Acad. Sci. U. S. A. 107 (33), 14662–14667. 10.1073/pnas.0909432107 20675583 PMC2930423

[B16] Ching Y. H. Ghosh T. K. Cross S. J. Packham E. A. Honeyman L. Loughna S. (2005). Mutation in myosin heavy chain 6 causes atrial septal defect. Nat. Genet. 37 (4), 423–428. 10.1038/ng1526 15735645

[B17] Connor J. A. Thiagarajan R. (2007). Hypoplastic left heart syndrome. Orphanet J. Rare Dis. 2, 23. 10.1186/1750-1172-2-23 17498282 PMC1877799

[B18] Costain G. Silversides C. K. Bassett A. S. (2016). The importance of copy number variation in congenital heart disease. NPJ Genom Med. 1, 16031. 10.1038/npjgenmed.2016.31 28706735 PMC5505728

[B19] Dannenberg J. H. David G. Zhong S. van der Torre J. Wong W. H. Depinho R. A. (2005). mSin3A corepressor regulates diverse transcriptional networks governing normal and neoplastic growth and survival. Genes Dev. 19 (13), 1581–1595. 10.1101/gad.1286905 15998811 PMC1172064

[B20] de Pater E. Clijsters L. Marques S. R. Lin Y. F. Garavito-Aguilar Z. V. Yelon D. (2009). Distinct phases of cardiomyocyte differentiation regulate growth of the zebrafish heart. Development 136 (10), 1633–1641. 10.1242/dev.030924 19395641 PMC2673760

[B21] Dobi K. C. Halfon M. S. Baylies M. K. (2014). Whole-genome analysis of muscle founder cells implicates the chromatin regulator Sin3A in muscle identity. Cell Rep. 8 (3), 858–870. 10.1016/j.celrep.2014.07.005 25088419 PMC4207094

[B22] Donlin L. T. Andresen C. Just S. Rudensky E. Pappas C. T. Kruger M. (2012). Smyd2 controls cytoplasmic lysine methylation of Hsp90 and myofilament organization. Genes Dev. 26 (2), 114–119. 10.1101/gad.177758.111 22241783 PMC3273835

[B23] Fahed A. C. Gelb B. D. Seidman J. G. Seidman C. E. (2013). Genetics of congenital heart disease: the glass half empty. Circ. Res. 112 (4), 707–720. 10.1161/CIRCRESAHA.112.300853 23410880 PMC3827691

[B24] Felker A. Prummel K. D. Merks A. M. Mickoleit M. Brombacher E. C. Huisken J. (2018). Continuous addition of progenitors forms the cardiac ventricle in zebrafish. Nat. Commun. 9 (1), 2001. 10.1038/s41467-018-04402-6 29784942 PMC5962599

[B25] Fleischer T. C. Yun U. J. Ayer D. E. (2003). Identification and characterization of three new components of the mSin3A corepressor complex. Mol. Cell Biol. 23 (10), 3456–3467. 10.1128/mcb.23.10.3456-3467.2003 12724404 PMC164750

[B26] Gabriel G. C. Devine W. Redel B. K. Whitworth K. M. Samuel M. Spate L. D. (2021). Cardiovascular development and congenital heart disease modeling in the pig. J. Am. Heart Assoc. 10 (14), e021631. 10.1161/JAHA.121.021631 34219463 PMC8483476

[B27] Gagnon J. A. Valen E. Thyme S. B. Huang P. Akhmetova L. Pauli A. (2014). Efficient mutagenesis by Cas9 protein-mediated oligonucleotide insertion and large-scale assessment of single-guide RNAs. PLoS One 9 (5), e98186. 10.1371/journal.pone.0098186 24873830 PMC4038517

[B28] Girstmair J. Zakrzewski A. Lapraz F. Handberg-Thorsager M. Tomancak P. Pitrone P. G. (2016). Light-sheet microscopy for everyone? Experience of building an OpenSPIM to study flatworm development. BMC Dev. Biol. 16 (1), 22. 10.1186/s12861-016-0122-0 27363495 PMC4929743

[B29] Granados-Riveron J. T. Ghosh T. K. Pope M. Bu'Lock F. Thornborough C. Eason J. (2010). Alpha-cardiac myosin heavy chain (MYH6) mutations affecting myofibril formation are associated with congenital heart defects. Hum. Mol. Genet. 19 (20), 4007–4016. 10.1093/hmg/ddq315 20656787

[B30] Grimes A. C. Erwin K. N. Stadt H. A. Hunter G. L. Gefroh H. A. Tsai H. J. (2008). PCB126 exposure disrupts zebrafish ventricular and branchial but not early neural crest development. Toxicol. Sci. 106 (1), 193–205. 10.1093/toxsci/kfn154 18660518 PMC2563148

[B31] Guner-Ataman B. Paffett-Lugassy N. Adams M. S. Nevis K. R. Jahangiri L. Obregon P. (2013). Zebrafish second heart field development relies on progenitor specification in anterior lateral plate mesoderm and nkx2.5 function. Development 140 (6), 1353–1363. 10.1242/dev.088351 23444361 PMC3585667

[B32] Hami D. Grimes A. C. Tsai H. J. Kirby M. L. (2011). Zebrafish cardiac development requires a conserved secondary heart field. Development 138 (11), 2389–2398. 10.1242/dev.061473 21558385 PMC3091499

[B33] Han P. Hang C. T. Yang J. Chang C. P. (2011). Chromatin remodeling in cardiovascular development and physiology. Circ. Res. 108 (3), 378–396. 10.1161/CIRCRESAHA.110.224287 21293009 PMC3079363

[B34] Hang C. T. Yang J. Han P. Cheng H. L. Shang C. Ashley E. (2010). Chromatin regulation by Brg1 underlies heart muscle development and disease. Nature 466 (7302), 62–67. 10.1038/nature09130 20596014 PMC2898892

[B35] Hargreaves D. C. Crabtree G. R. (2011). ATP-Dependent chromatin remodeling: genetics, genomics and mechanisms. Cell Res. 21 (3), 396–420. 10.1038/cr.2011.32 21358755 PMC3110148

[B36] Haverinen J. Hassinen M. Dash S. N. Vornanen M. (2018). Expression of calcium channel transcripts in the zebrafish heart: dominance of T-type channels. J. Exp. Biol. 221 (10), jeb179226. 10.1242/jeb.179226 29739832

[B37] Hota S. K. Johnson J. R. Verschueren E. Thomas R. Blotnick A. M. Zhu Y. (2019). Dynamic BAF chromatin remodeling complex subunit inclusion promotes temporally distinct gene expression programs in cardiogenesis. Development 146 (19), dev174086. 10.1242/dev.174086 30814119 PMC6803373

[B38] Huang C. J. Tu C. T. Hsiao C. D. Hsieh F. J. Tsai H. J. (2003). Germ-line transmission of a myocardium-specific GFP transgene reveals critical regulatory elements in the cardiac myosin light chain 2 promoter of zebrafish. Dev. Dyn. 228 (1), 30–40. 10.1002/dvdy.10356 12950077

[B39] Huang H. T. Kathrein K. L. Barton A. Gitlin Z. Huang Y. H. Ward T. P. (2013). A network of epigenetic regulators guides developmental haematopoiesis *in vivo* . Nat. Cell Biol. 15 (12), 1516–1525. 10.1038/ncb2870 24240475 PMC3959952

[B40] Huang M. Akerberg A. A. Zhang X. Yoon H. Joshi S. Hallinan C. (2022). Intrinsic myocardial defects underlie an Rbfox-deficient zebrafish model of hypoplastic left heart syndrome. Nat. Commun. 13 (1), 5877. 10.1038/s41467-022-32982-x 36198703 PMC9534849

[B41] Ignatius M. S. Unal Eroglu A. Malireddy S. Gallagher G. Nambiar R. M. Henion P. D. (2013). Distinct functional and temporal requirements for zebrafish Hdac1 during neural crest-derived craniofacial and peripheral neuron development. PLoS One 8 (5), e63218. 10.1371/journal.pone.0063218 23667588 PMC3646935

[B42] Itou J. Oishi I. Kawakami H. Glass T. J. Richter J. Johnson A. (2012). Migration of cardiomyocytes is essential for heart regeneration in zebrafish. Development 139 (22), 4133–4142. 10.1242/dev.079756 23034636

[B43] Jensen B. Wang T. Christoffels V. M. Moorman A. F. (2013). Evolution and development of the building plan of the vertebrate heart. Biochim. Biophys. Acta 1833 (4), 783–794. 10.1016/j.bbamcr.2012.10.004 23063530

[B44] Jiang D. Jiang Z. Lu D. Wang X. Liang H. Zhang J. (2019). Migrasomes provide regional cues for organ morphogenesis during zebrafish gastrulation. Nat. Cell Biol. 21 (8), 966–977. 10.1038/s41556-019-0358-6 31371827

[B45] Jing L. (2012). Zebrafish embryo DNA preparation. Bio-protocol 2, e184. 10.21769/bioprotoc.184

[B46] Jones W. K. Grupp I. L. Doetschman T. Grupp G. Osinska H. Hewett T. E. (1996). Ablation of the murine alpha myosin heavy chain gene leads to dosage effects and functional deficits in the heart. J. Clin. Invest. 98 (8), 1906–1917. 10.1172/JCI118992 8878443 PMC507631

[B47] Joshi A. A. Struhl K. (2005). Eaf3 chromodomain interaction with methylated H3-K36 links histone deacetylation to Pol II elongation. Mol. Cell 20 (6), 971–978. 10.1016/j.molcel.2005.11.021 16364921

[B48] Kadamb R. Mittal S. Bansal N. Batra H. Saluja D. (2013). Sin3: insight into its transcription regulatory functions. Eur. J. Cell Biol. 92 (8-9), 237–246. 10.1016/j.ejcb.2013.09.001 24189169

[B49] Keogh M. C. Kurdistani S. K. Morris S. A. Ahn S. H. Podolny V. Collins S. R. (2005). Cotranscriptional set2 methylation of histone H3 lysine 36 recruits a repressive Rpd3 complex. Cell 123 (4), 593–605. 10.1016/j.cell.2005.10.025 16286008

[B50] Knight H. G. Yelon D. (2016). Utilizing zebrafish to understand second heart field development. 10.1007/978-4-431-54628-3_25 29787152

[B51] Lagger G. O'Carroll D. Rembold M. Khier H. Tischler J. Weitzer G. (2002). Essential function of histone deacetylase 1 in proliferation control and CDK inhibitor repression. EMBO J. 21 (11), 2672–2681. 10.1093/emboj/21.11.2672 12032080 PMC126040

[B52] Lazic S. Scott I. C. (2011). Mef2cb regulates late myocardial cell addition from a second heart field-like population of progenitors in zebrafish. Dev. Biol. 354 (1), 123–133. 10.1016/j.ydbio.2011.03.028 21466801

[B53] Lei I. Gao X. Sham M. H. Wang Z. (2012). SWI/SNF protein component BAF250a regulates cardiac progenitor cell differentiation by modulating chromatin accessibility during second heart field development. J. Biol. Chem. 287 (29), 24255–24262. 10.1074/jbc.M112.365080 22621927 PMC3397851

[B54] Lei I. Tian S. Chen V. Zhao Y. Wang Z. (2019). SWI/SNF component BAF250a coordinates OCT4 and WNT signaling pathway to control cardiac lineage differentiation. Front. Cell Dev. Biol. 7, 358. 10.3389/fcell.2019.00358 32039194 PMC6987383

[B55] Lickert H. Takeuchi J. K. Von Both I. Walls J. R. McAuliffe F. Adamson S. L. (2004). Baf60c is essential for function of BAF chromatin remodelling complexes in heart development. Nature 432 (7013), 107–112. 10.1038/nature03071 15525990

[B56] Lifschitz S. Haeusler E. H. Catanho M. Miranda A. B. Armas E. M. Heine A. (2022). Bio-strings: A relational database data-type for dealing with large biosequences. Biotech. (Basel). 11 (3), 31. 10.3390/biotech11030031 35997339 PMC9472027

[B57] Liu X. Yagi H. Saeed S. Bais A. S. Gabriel G. C. Chen Z. (2017). The complex genetics of hypoplastic left heart syndrome. Nat. Genet. 49 (7), 1152–1159. 10.1038/ng.3870 28530678 PMC5737968

[B58] Martinez S. R. Gay M. S. Zhang L. (2015). Epigenetic mechanisms in heart development and disease. Drug Discov. Today 20 (7), 799–811. 10.1016/j.drudis.2014.12.018 25572405 PMC4492921

[B59] McKinsey T. A. (2011). The biology and therapeutic implications of HDACs in the heart. Handb. Exp. Pharmacol. 206, 57–78. 10.1007/978-3-642-21631-2_4 21879446

[B60] Monserrat L. Hermida-Prieto M. Fernandez X. Rodriguez I. Dumont C. Cazon L. (2007). Mutation in the alpha-cardiac actin gene associated with apical hypertrophic cardiomyopathy, left ventricular non-compaction, and septal defects. Eur. Heart J. 28 (16), 1953–1961. 10.1093/eurheartj/ehm239 17611253

[B61] Montgomery R. L. Davis C. A. Potthoff M. J. Haberland M. Fielitz J. Qi X. (2007). Histone deacetylases 1 and 2 redundantly regulate cardiac morphogenesis, growth, and contractility. Genes Dev. 21 (14), 1790–1802. 10.1101/gad.1563807 17639084 PMC1920173

[B62] Morano I. Chai G. X. Baltas L. G. Lamounier-Zepter V. Lutsch G. Kott M. (2000). Smooth-muscle contraction without smooth-muscle myosin. Nat. Cell Biol. 2 (6), 371–375. 10.1038/35014065 10854329

[B63] Mortensen S. A. Skov L. L. Kjaer-Sorensen K. Hansen A. G. Hansen S. Dagnaes-Hansen F. (2017). Endogenous natural complement inhibitor regulates cardiac development. J. Immunol. 198 (8), 3118–3126. 10.4049/jimmunol.1601958 28258200

[B64] Nakamura R. Koshiba-Takeuchi K. Tsuchiya M. Kojima M. Miyazawa A. Ito K. (2016). Expression analysis of Baf60c during heart regeneration in axolotls and neonatal mice. Dev. Growth Differ. 58 (4), 367–382. 10.1111/dgd.12281 27125315

[B65] Nambiar R. M. Ignatius M. S. Henion P. D. (2007). Zebrafish colgate/hdac1 functions in the non-canonical Wnt pathway during axial extension and in Wnt-independent branchiomotor neuron migration. Mech. Dev. 124 (9-10), 682–698. 10.1016/j.mod.2007.07.003 17716875 PMC2701655

[B66] Nora J. J. (1968). Multifactorial inheritance hypothesis for the etiology of congenital heart diseases. The genetic-environmental interaction. Circulation 38 (3), 604–617. 10.1161/01.cir.38.3.604 4876982

[B67] Papa A. Kushner J. Marx S. O. (2022). Adrenergic regulation of calcium channels in the heart. Annu. Rev. Physiol. 84, 285–306. 10.1146/annurev-physiol-060121-041653 34752709 PMC9573788

[B68] Pitrone P. G. Schindelin J. Stuyvenberg L. Preibisch S. Weber M. Eliceiri K. W. (2013). OpenSPIM: an open-access light-sheet microscopy platform. Nat. Methods 10 (7), 598–599. 10.1038/nmeth.2507 23749304 PMC7450513

[B69] Postma A. V. van Engelen K. van de Meerakker J. Rahman T. Probst S. Baars M. J. (2011). Mutations in the sarcomere gene MYH7 in Ebstein anomaly. Circ. Cardiovasc Genet. 4 (1), 43–50. 10.1161/CIRCGENETICS.110.957985 21127202

[B70] Pyo R. T. Sui J. Dhume A. Palomeque J. Blaxall B. C. Diaz G. (2006). CXCR4 modulates contractility in adult cardiac myocytes. J. Mol. Cell Cardiol. 41 (5), 834–844. 10.1016/j.yjmcc.2006.08.008 17010372 PMC2002477

[B71] R Core Team (2021). R: A language and environment for statistical computing. Vienna, Austria: R Foundation for Statistical Computing. https://www.R-project.org/ .

[B72] Revell L. J. Graham Reynolds R. (2012). A new Bayesian method for fitting evolutionary models to comparative data with intraspecific variation. Evolution 66 (9), 2697–2707. 10.1111/j.1558-5646.2012.01645.x 22946797

[B73] Robinson M. D. McCarthy D. J. Smyth G. K. (2010). edgeR: a Bioconductor package for differential expression analysis of digital gene expression data. Bioinformatics 26 (1), 139–140. 10.1093/bioinformatics/btp616 19910308 PMC2796818

[B74] Severs N. J. Dupont E. Coppen S. R. Halliday D. Inett E. Baylis D. (2004). Remodelling of gap junctions and connexin expression in heart disease. Biochim. Biophys. Acta 1662 (1-2), 138–148. 10.1016/j.bbamem.2003.10.019 15033584

[B75] Shah K. Seeley S. Schulz C. Fisher J. Gururaja Rao S. (2022). Calcium channels in the heart: disease States and drugs. Cells 11 (6), 943. 10.3390/cells11060943 35326393 PMC8945986

[B76] Sherman B. T. Hao M. Qiu J. Jiao X. Baseler M. W. Lane H. C. (2022). David: A web server for functional enrichment analysis and functional annotation of gene lists (2021 update). Nucleic Acids Res. 50 (W1), W216–W221. 10.1093/nar/gkac194 35325185 PMC9252805

[B77] Singh A. P. Archer T. K. (2014). Analysis of the SWI/SNF chromatin-remodeling complex during early heart development and BAF250a repression cardiac gene transcription during P19 cell differentiation. Nucleic Acids Res. 42 (5), 2958–2975. 10.1093/nar/gkt1232 24335282 PMC3950667

[B78] Song Y. C. Dohn T. E. Rydeen A. B. Nechiporuk A. V. Waxman J. S. (2019). HDAC1-mediated repression of the retinoic acid-responsive gene ripply3 promotes second heart field development. PLoS Genet. 15 (5), e1008165. 10.1371/journal.pgen.1008165 31091225 PMC6538190

[B79] Stankunas K. Hang C. T. Tsun Z. Y. Chen H. Lee N. V. Wu J. I. (2008). Endocardial Brg1 represses ADAMTS1 to maintain the microenvironment for myocardial morphogenesis. Dev. Cell 14 (2), 298–311. 10.1016/j.devcel.2007.11.018 18267097 PMC2274005

[B80] Sun X. Hota S. K. Zhou Y. Q. Novak S. Miguel-Perez D. Christodoulou D. (2018). Cardiac-enriched BAF chromatin-remodeling complex subunit Baf60c regulates gene expression programs essential for heart development and function. Biol. Open 7 (1), bio029512. 10.1242/bio.029512 29183906 PMC5829499

[B81] Takeuchi J. K. Lou X. Alexander J. M. Sugizaki H. Delgado-Olguin P. Holloway A. K. (2011). Chromatin remodelling complex dosage modulates transcription factor function in heart development. Nat. Commun. 2, 187. 10.1038/ncomms1187 21304516 PMC3096875

[B82] Trotter K. W. Archer T. K. (2008). The BRG1 transcriptional coregulator. Nucl. Recept Signal 6, e004. 10.1621/nrs.06004 18301784 PMC2254329

[B83] van Engelen K. Postma A. V. van de Meerakker J. B. Roos-Hesselink J. W. Helderman-van den Enden A. T. Vliegen H. W. (2013). Ebstein's anomaly may be caused by mutations in the sarcomere protein gene MYH7. Neth Heart J. 21 (3), 113–117. 10.1007/s12471-011-0141-1 21604106 PMC3578524

[B84] van Oevelen C. Bowman C. Pellegrino J. Asp P. Cheng J. Parisi F. (2010). The mammalian Sin3 proteins are required for muscle development and sarcomere specification. Mol. Cell Biol. 30 (24), 5686–5697. 10.1128/MCB.00975-10 20956564 PMC3004272

[B85] van Oevelen C. Wang J. Asp P. Yan Q. Kaelin W. G. Jr. Kluger Y. (2008). A role for mammalian Sin3 in permanent gene silencing. Mol. Cell 32 (3), 359–370. 10.1016/j.molcel.2008.10.015 18995834 PMC3100182

[B86] Voelkel T. Andresen C. Unger A. Just S. Rottbauer W. Linke W. A. (2013). Lysine methyltransferase Smyd2 regulates Hsp90-mediated protection of the sarcomeric titin springs and cardiac function. Biochim. Biophys. Acta 1833 (4), 812–822. 10.1016/j.bbamcr.2012.09.012 23047121

[B87] Wang Z. Zhai W. Richardson J. A. Olson E. N. Meneses J. J. Firpo M. T. (2004). Polybromo protein BAF180 functions in mammalian cardiac chamber maturation. Genes Dev. 18 (24), 3106–3116. 10.1101/gad.1238104 15601824 PMC535920

[B88] Wickham H. (2016). ggplot2: Elegant graphics for data analysis. New York: Springer-Verlag.

[B89] Xiao C. Gao L. Hou Y. Xu C. Chang N. Wang F. (2016). Chromatin-remodelling factor Brg1 regulates myocardial proliferation and regeneration in zebrafish. Nat. Commun. 7, 13787. 10.1038/ncomms13787 27929112 PMC5476829

[B90] Xiao D. Wang H. Hao L. Guo X. Ma X. Qian Y. (2018). The roles of SMYD4 in epigenetic regulation of cardiac development in zebrafish. PLoS Genet. 14 (8), e1007578. 10.1371/journal.pgen.1007578 30110327 PMC6110521

[B91] Yalcin H. C. Amindari A. Butcher J. T. Althani A. Yacoub M. (2017). Heart function and hemodynamic analysis for zebrafish embryos. Dev. Dyn. 246 (11), 868–880. 10.1002/dvdy.24497 28249360

[B92] Yu G. Lam T. T. Zhu H. Guan Y. (2018). Two methods for mapping and visualizing associated data on phylogeny using ggtree. Mol. Biol. Evol. 35 (12), 3041–3043. 10.1093/molbev/msy194 30351396 PMC6278858

[B93] Yu G. (2020). Using ggtree to visualize data on tree-like structures. Curr. Protoc. Bioinforma. 69 (1), e96. 10.1002/cpbi.96 32162851

[B94] Zhu F. Zhu Q. Ye D. Zhang Q. Yang Y. Guo X. (2018). Sin3a-Tet1 interaction activates gene transcription and is required for embryonic stem cell pluripotency. Nucleic Acids Res. 46 (12), 6026–6040. 10.1093/nar/gky347 29733394 PMC6158608

[B95] Zhu L. Vranckx R. Khau Van Kien P. Lalande A. Boisset N. Mathieu F. (2006). Mutations in myosin heavy chain 11 cause a syndrome associating thoracic aortic aneurysm/aortic dissection and patent ductus arteriosus. Nat. Genet. 38 (3), 343–349. 10.1038/ng1721 16444274

[B96] Znosko W. A. Yu S. Thomas K. Molina G. A. Li C. Tsang W. (2010). Overlapping functions of Pea3 ETS transcription factors in FGF signaling during zebrafish development. Dev. Biol. 342 (1), 11–25. 10.1016/j.ydbio.2010.03.011 20346941 PMC2866755

