# Supplementary material for: Sin3a associated protein 130 kDa, sap130, plays an evolutionary conserved role in zebrafish heart development
Source: Front Cell Dev Biol. 2023 Aug 30;11:1197109. doi: 10.3389/fcell.2023.1197109 (PMC10498550; doi:10.3389/fcell.2023.1197109)

Sin3a Associated Protein 130kDa, sap130, plays an evolutionary conserved role in zebrafish heart development

Ricardo A. DeMoya[1], Rachel E. Forman-Rubinsky[1], Deon Fontaine Jr.[1], Joseph Shin[1], Simon C. Watkins[2], Cecilia Lo[1] and Michael Tsang[1]

[1]Department of Developmental Biology, University of Pittsburgh, School of Medicine, Pittsburgh PA 15213, USA

[2]Department of Cell Biology and Molecular Physiology, University of Pittsburgh School of Medicine, Pittsburgh, PA, 15261, USA

†To whom correspondence may be addressed. Email: tsang@pitt.edu

Supplemental Figures

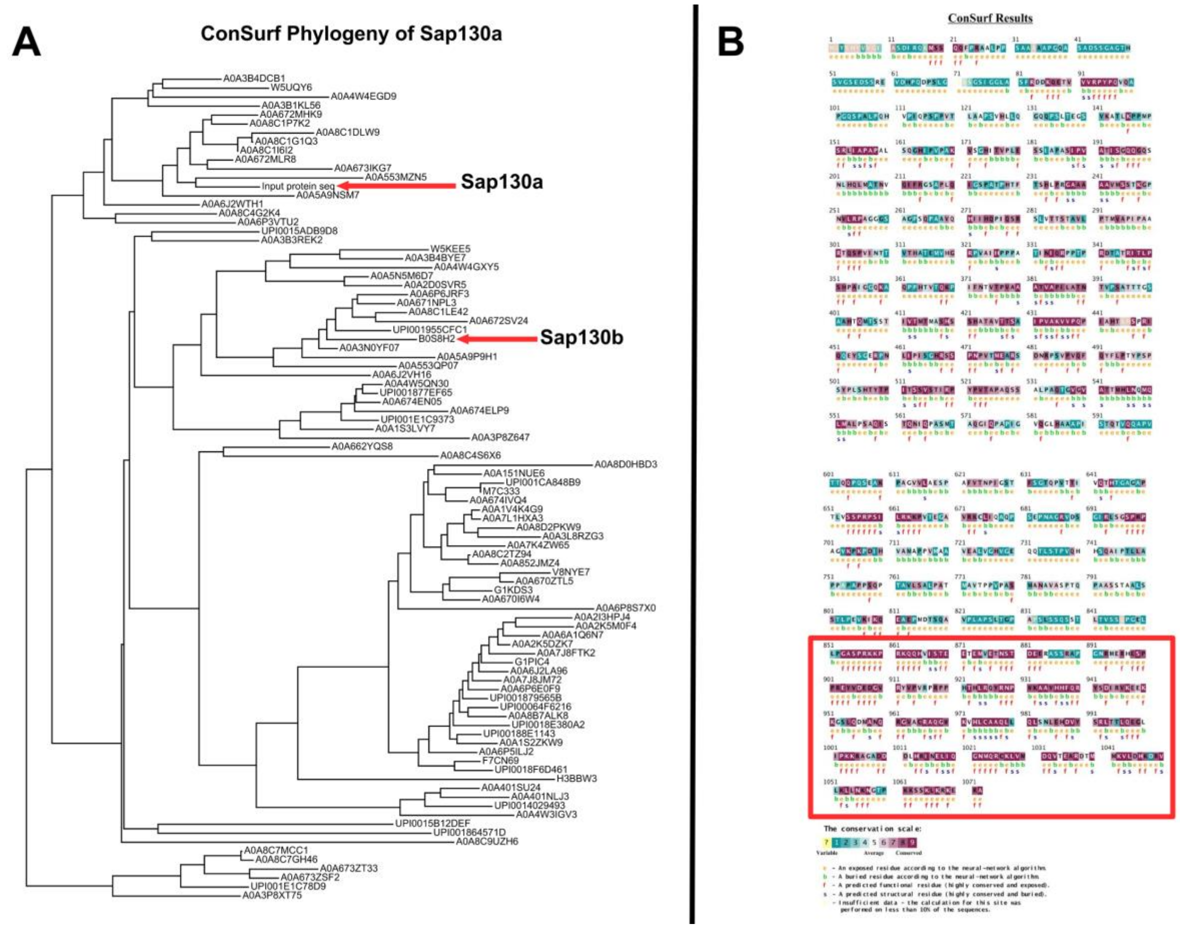

Figure S1: ConSurf output from Sap130a protein sequence
(**A**) A phylogenetic tree with UniProt IDs made using ConSurf showing 145 unique sequences for zebrafish sap130a. (**B**) The ConSurf output for protein residue classification, Darker red meaning more likely conserved. Red Box outlines the conserved C-terminal domain with many dark red amino acids.

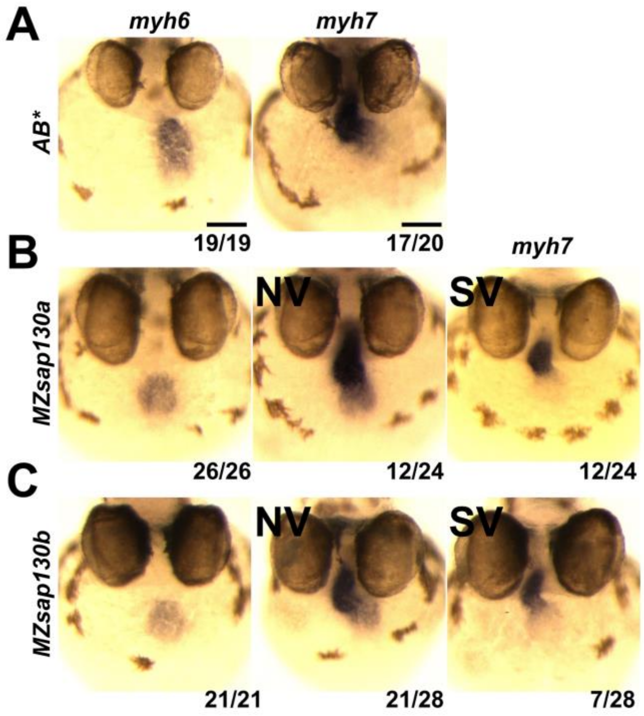

Figure S2: WISH at 48hpf of *MZsap130a* mutants have SVs
(**A, B, C**) WISH of 48hpf embryos for *myh6* (atria) and *myh7* (ventricle) of AB* and *MZsap130a* and *MZsap130b* mutants. Scale bar 100µm

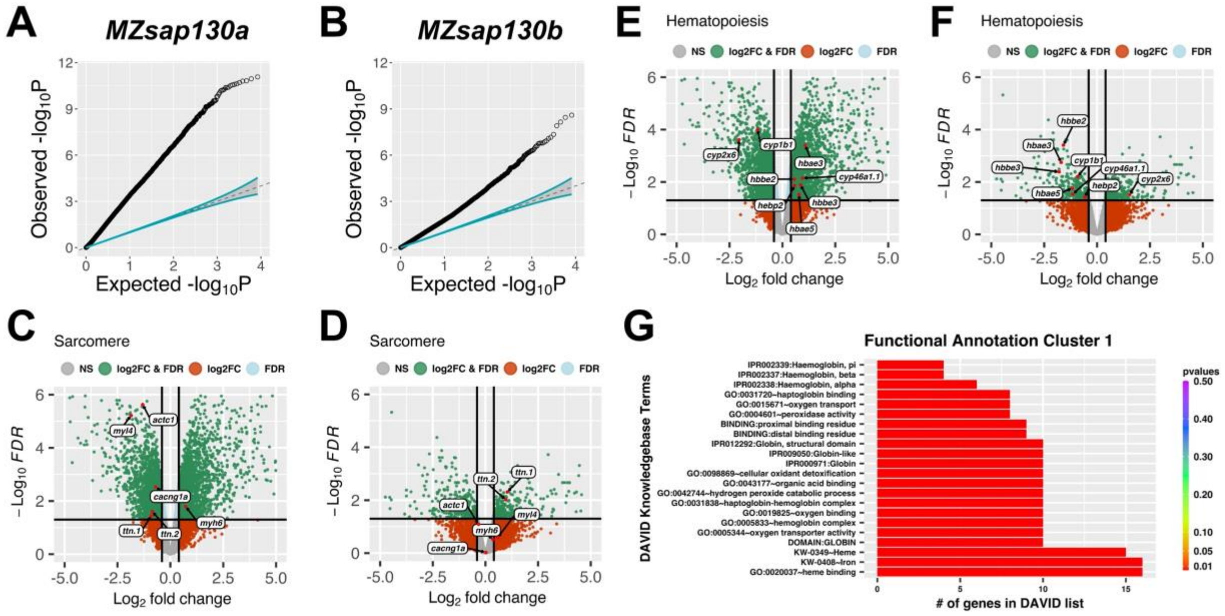

Figure S3: *MZsap130a* and *MZsap130b* show different gene expression profiles in zebrafish (**A, B**) Show the expected (in blue) and observed –log10(pvals) for *MZsap130a* and *MZsap130b* mutants 36hpf whole embryo RNAseq. (**C, D**) Volcano plots showing changes in sarcomere genes in *MZsap130a* and *MZsap130b*. (**E ,F**) Volcano plots showing DEGs that overlap between *MZsap130a* and *MZsap130b*, involved in hematopoiesis. (**G**) Shows the DAVID annotation cluster 1 of the overlap genes revealing hematopoietic gene groups.

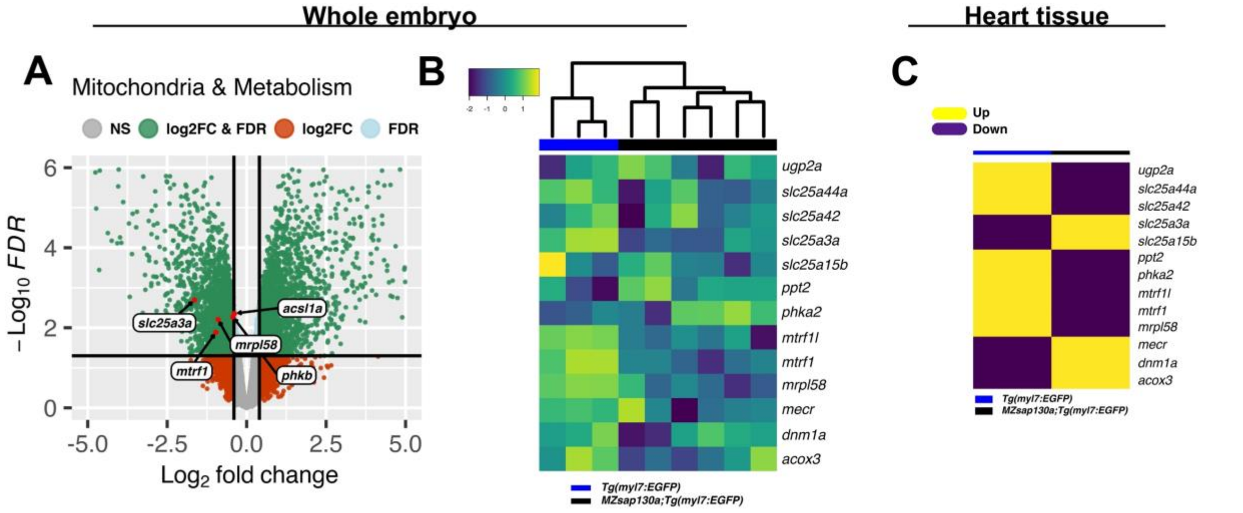

Figure S4: RNA-seq reveals DEGs and pathways in *MZsap130a* mutants
(**A, B**) Volcano plot and heatmap of genes associated with mitochondria and metabolism from whole embryo RNAseq at 36hpf. (**C**) Heatmap of the same genes in panels B, but for 48hpf heart tissue RNAseq. For 36hpf data an ANOVA-like analysis was used and for 48hpf heart tissue data we used a likelihood-ration test in edgeR.

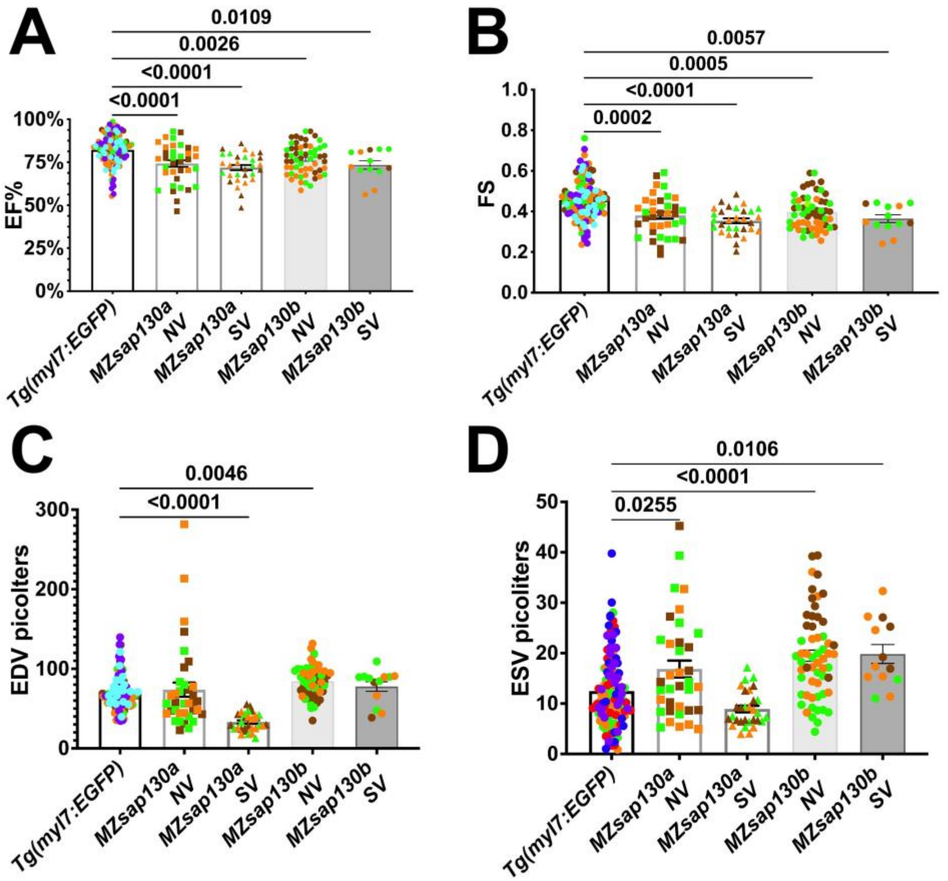

Figure S5: Cardiac parameters measured in *MZsap130* mutants
(**A, B, C, D**) Shows the cardiac parameters measured ejection fraction (EF), fractional shortening (FS), end-diastolic volume and end-systolic volumes (EDV and ESV). Each point represents individual ventricle and color coded for 3+ experiments. For *Tg(myl7:EGFP)*, n=115; *MZsap130a* NV, n=36; *MZsap130a* SV, n=30; *MZsap130b* NV, n=57; *MZsap130b* SV, n=13.

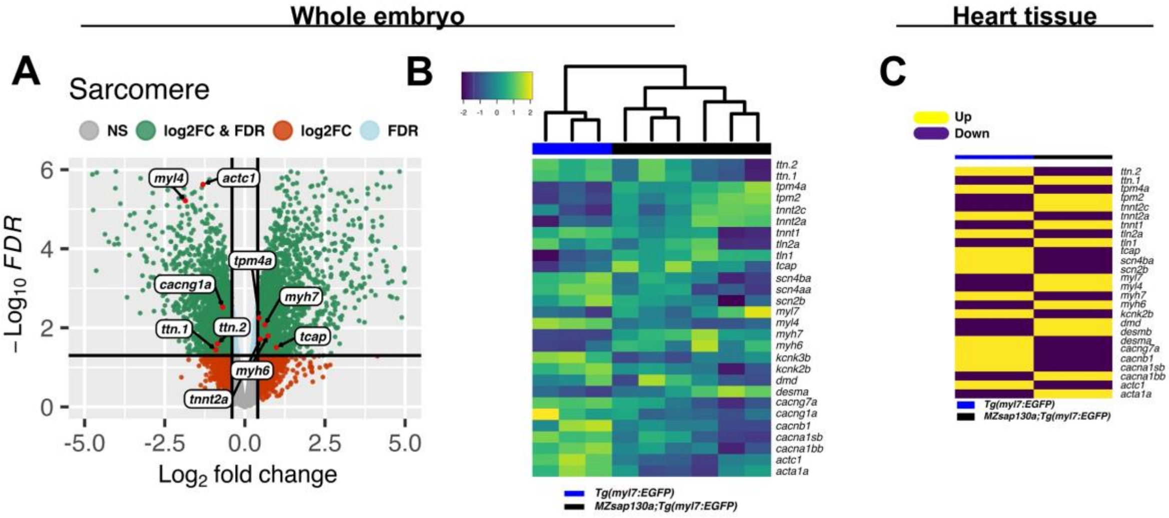

Figure S6: RNA-seq reveals DEGs and pathways in *MZsap130a* mutants
(**A, B**) Volcano plot and heatmap of genes associated with the sarcomere from whole embryo RNAseq at 36hpf. (**C**) Heatmap of the same genes in panels B, but for 48hpf heart tissue RNAseq. For 36hpf data an ANOVA-like analysis was used and for 48hpf heart tissue data we used a likelihood-ration test in edgeR.

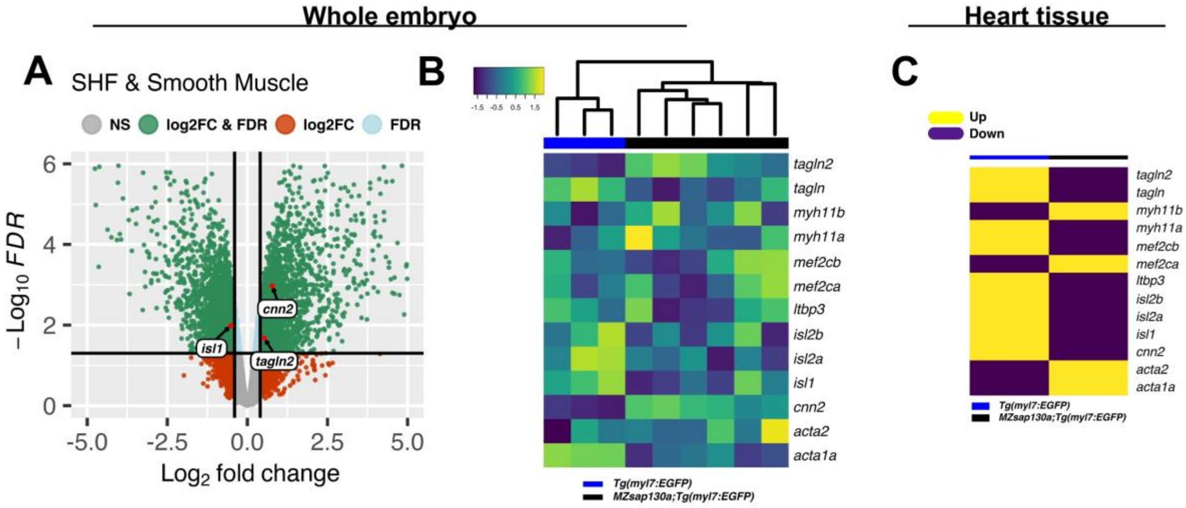

Figure S7: RNA-seq reveals DEGs and pathways in *MZsap130a* mutants
(**A, B**) Volcano plot and heatmap of genes associated with the SHF and smooth muscle from whole embryo RNAseq at 36hpf. (**C**) Heatmap of the same genes in panels B, but for 48hpf heart tissue RNAseq. For 36hpf data an ANOVA-like analysis was used and for 48hpf heart tissue data we used a likelihood-ration test in edgeR.

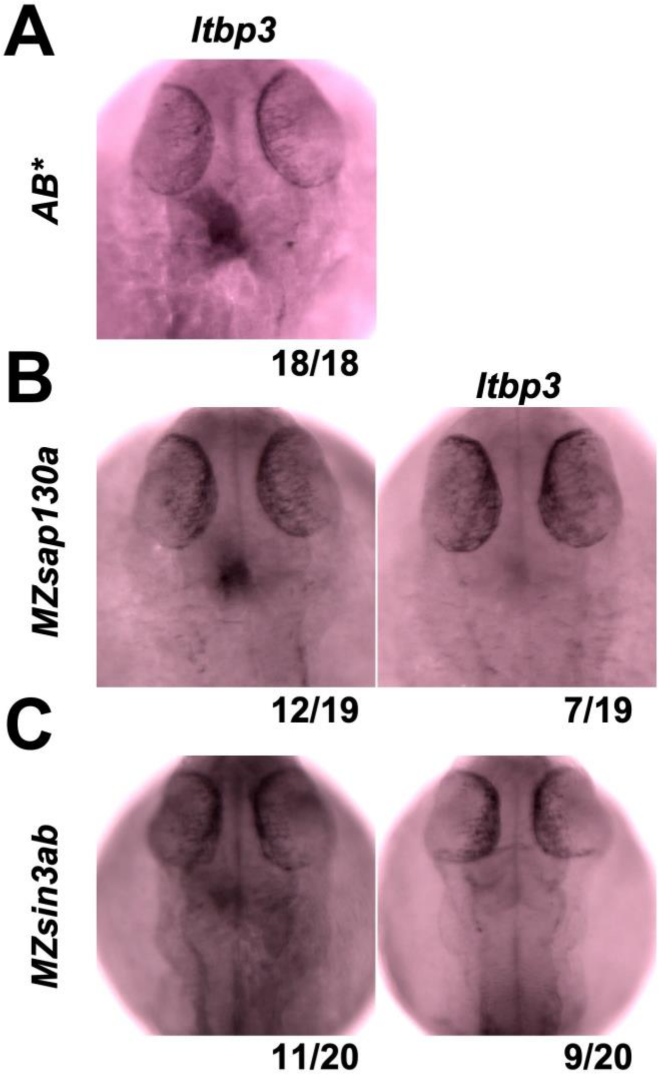

Figure S8: 30hpf *ltbp3* expression in *MZsap130a* and *MZsin3ab*
(A, B, C) *AB**, *MZsap130a*, *MZsin3ab* WISH *ltbp3* expression at 30hpf. Observed a reduction of *ltbp3* in both mutants.

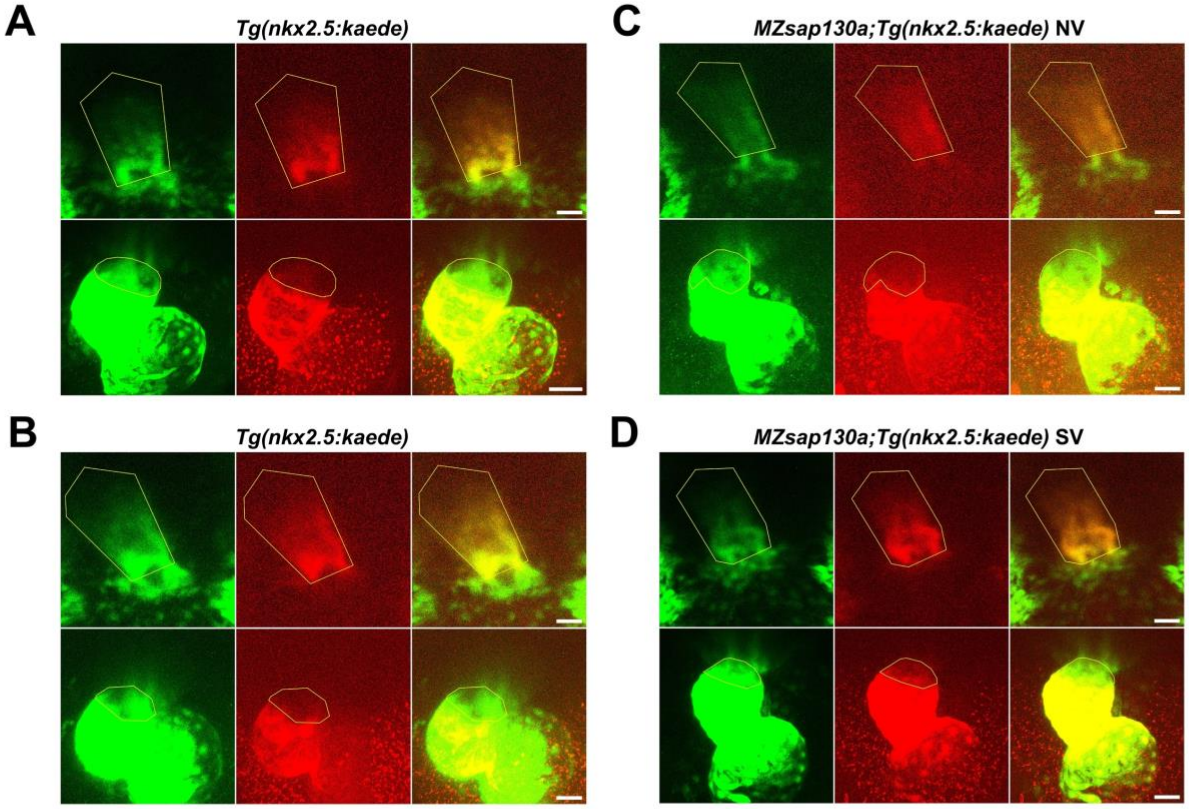

Figure S9: 24hpf heart tube photoconversion and 48hpf imaging and measuring examples (**A, B**) Shows examples of *Tg(nkx2.5:kaede)* being photoconverted at 24hpf and imaged at 48hpf. (**C, D**) Reveals another example of NV and SV *MZsap130a;Tg(nkx2.5:kaede)* mutants photoconverted at 24hpf and imaged at 48hpf. Yellow lines outline photoconverted region and the green area measured as SHF. *Tg(nkx2.5:kaede)*, n=15; *MZsap130a;Tg(nkx2.5:kaede)* NV, n=14; *MZsap130a;Tg(nkx2.5:kaede)* SV, n=10. Scale bar 50µm

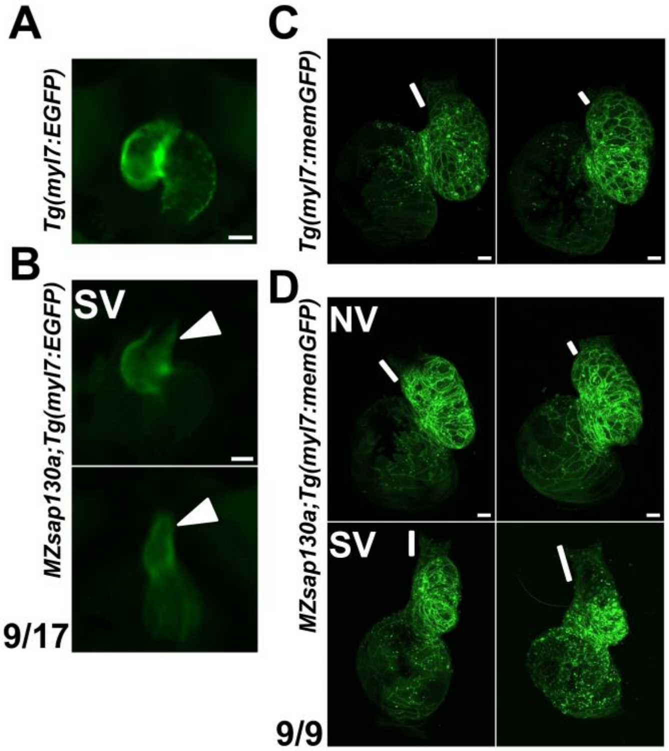

Figure S10: ***MZsap130a*** **OFT images at 48-72hpf**
(**A, B**) *Tg(myl7:EGFP*) and *MZsap130a;Tg(myl7:EGFP)* embryos at 48hpf with longer OFTs.
(**C, D**) *Tg(myl7:memGFP*) and *MZsap130a;Tg(myl7:memGFP)* embryos at 72hpf with longer OFTs. White triangles and lines highlight OFT structures. Scale bar = 50µm

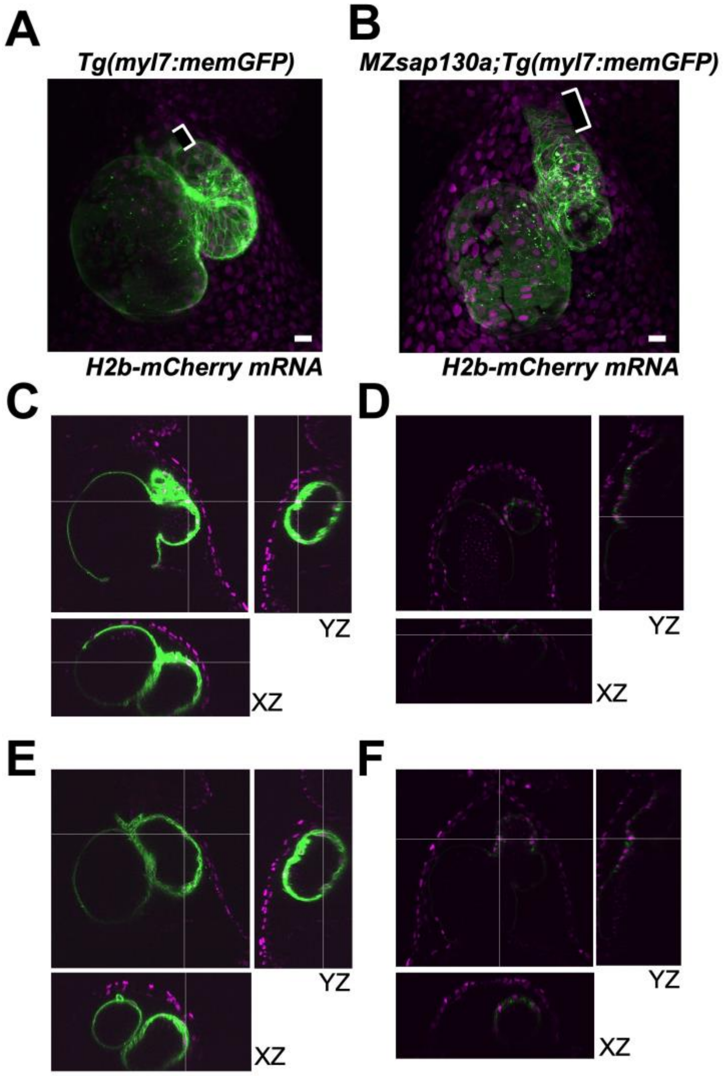

Figure S11: Counting cells in *MZsap130a* mutant ventricles and OFTs

(A) An average projection of *Tg(myl7:memGFP)* at 48hpf injected with *H2b:mCherry* mRNA at the one cell stage. (B) An average projection of *MZsap130a;Tg(myl7:memGFP)* at 48hpf injected with *H2b:mCherry* mRNA at the one cell stage. (C, E) Example slices for counting *Tg(myl7:memGFP)+ H2b:mCherry+* CMs, with orthogonal views. (D, F) Example slices for counting *MZsap130a;Tg(myl7:memGFP)+ H2b:mCherry+* CMs, with orthogonal views.

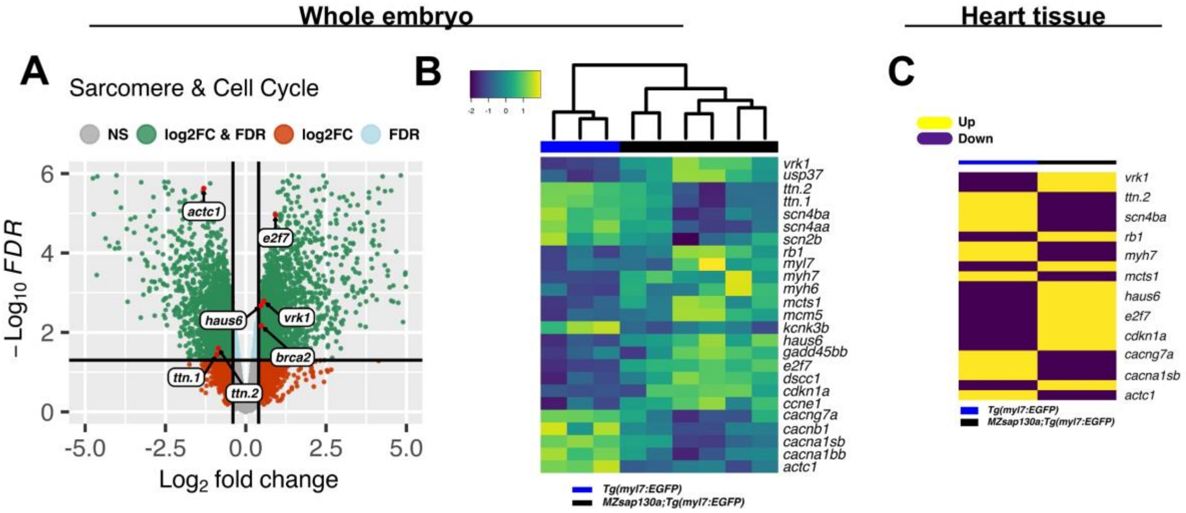

Figure S12: RNA-seq reveals DEGs and pathways in *MZsap130a* mutants
(**A, B**) Volcano plot and heatmap of genes associated with the sarcomere and cell cycle from whole embryo RNAseq at 36hpf. (**C**) Heatmap of the same genes in panels B, but for 48hpf heart tissue RNAseq. For 36hpf data an ANOVA-like analysis was used and for 48hpf heart tissue data we used a likelihood-ration test in edgeR.

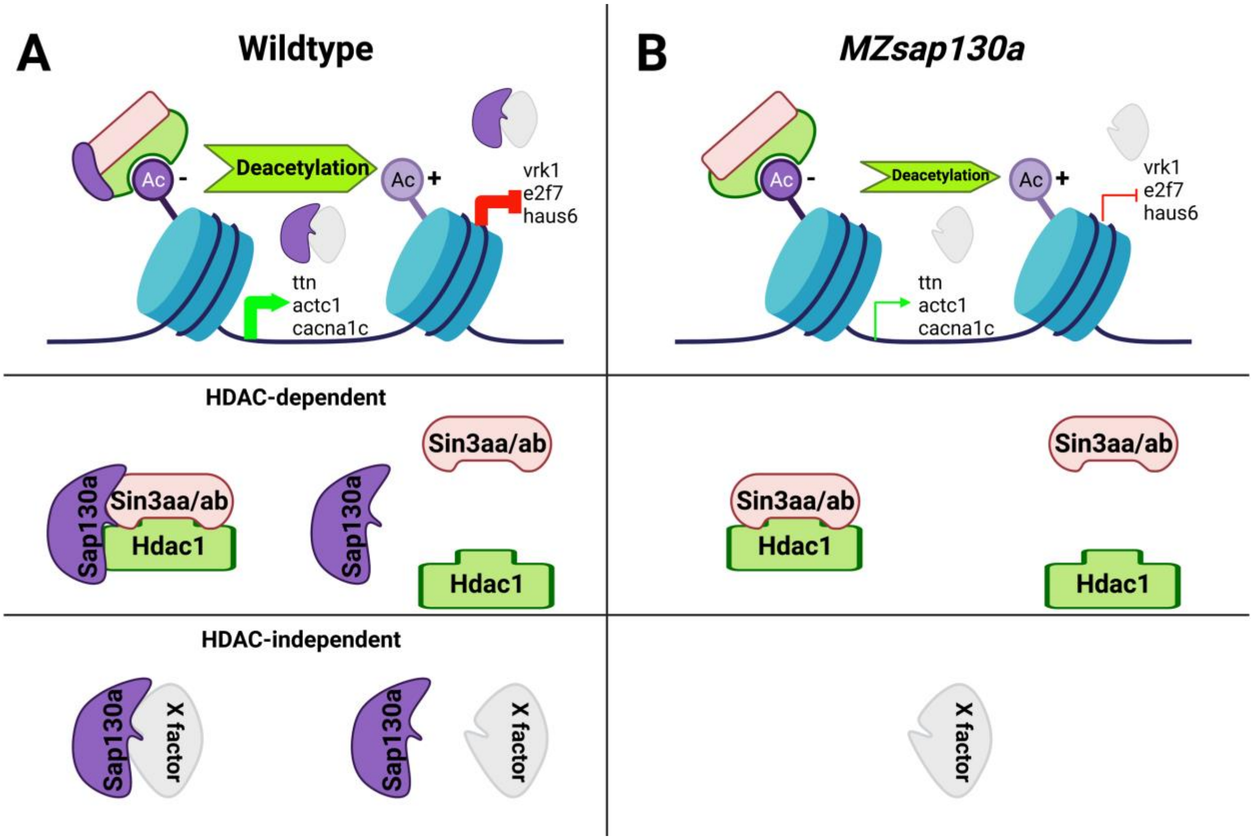

Figure S13: Proposed role for *sap130a* in *sin3ab* and *hdac1* complex in zebrafish
(**A**) Shows wildtype setting with Sap130a present interacting with the Sin3aa/ab Hdac1 complex or an unknown X factor, to account for HDAC-independent functions. (**B**) Shows the possibilities when Sap130a is missing. Genes shown to change can be found in **Figure S12**

WT vs *MZsap130a* 36hpf

Ricardo A. DeMoya

2023-06-18

RNAseq analysis pipeline

The csv file that contains the count data to b uploaded, is from total RNA collected from whole embryos at 36hpf for WT and *MZsap130a* genotypes. The *MZsap130a* animals were further separated into the normal ventricle (NV) or small ventricle (SV) groups for RNA extraction. The total RNA was sent to sequencing core at the University of Pittsburgh with three pooled biological replicates for each group (WT1-3, NV1-3, and SV1-3, ~75 embryos per sample). Once the *fastq* files were obtain we uploaded them in CLC Genomics Workbench by QIAGEN and used the RNAseq analysis tool to produce a Total count matrix for export as a csv file. This file was then used in R and Rstudio to explore the relationship between WT and *MZsap130a* mutants.

Data input and metadata

Using the read.table() function, we import the data table from a csv count matrix generated by CLC Gx workbench. Other alignment methods can be used including, Rbowtie2, TopHat2, Rsubread, or HISAT2. Annotation was done using GRCz11 zebrafish reference genome.

Read in the expression count matrix, exported from CLC Gx after annotation is done with GRCz11

```
data_edger = read.table(
  file="data/processed_data/MZsap130a_pt32a_36hpf_RNAseq_count_matrix.csv",
  sep=",",
  header=T)
head(data_edger[,1:4], n = 5)
```

```
##              Feature.ID        ENSEMBL.IDs AB_1_36hpf AB_3_36hpf
## 1                  a1cf ENSDARG00000002968        295        361
## 2                  a2ml ENSDARG00000056314       6828       8808
## 3 A2ML1 (1 of many)_1 ENSDARG00000115205         14          9
## 4 A2ML1 (1 of many)_2 ENSDARG00000008835          1          4
## 5 A2ML1 (1 of many)_3 ENSDARG00000041645       2227       2480
```

Updating gene names from GRCz11

```
data_edger$Feature.ID[data_edger$Feature.ID == "ACTC1"] <- "actc1"
data_edger$Feature.ID[data_edger$Feature.ID == "FP102018.1"] <- "ltbp3"
```

Making a data frame and setting the rownames to genenames or ENSEMBL IDs

```
data_edger <- data.frame(data_edger, row.names = data_edger$Feature.ID)
```

Double checking for duplicates

```
# Makes a list of ENSEMBL IDs in the data set and makes sure there are no duplicates
unique_edger = names(which(table(as.vector(data_edger[,2])) == 1))
# Indexes your data for rows that are present in the list you made in the last step
unique.data_edger = data_edger[which(data_edger[,2] %in% unique_edger),]

# Repackage as a dataframe
unique.data_edger = data.frame(unique.data_edger)
# This makes the rownames equal to genenames
rownames(unique.data_edger) = unique.data_edger[,1] # change to 2 for IDs
# This will remove the ID and names columns in this data set column 1 and 2
unique.data_edger = unique.data_edger[,-1:-2]
```

Creating a metadata table

```
# Creates a design matrix for modeling the sequencing experiment and any co-variants
# WT, NV, SV all separate
coldat <- data.frame(row.names = colnames(unique.data_edger), stringsAsFactors = T)
coldat$Groups <- factor(c(rep("WT", 3), rep("NV", 3), rep("SV",3)),
                        levels = c("WT","NV","SV"))
coldat$Genotype <- factor(c(rep("AB*",3),rep("sap130a^pt32a/pt32a", 6)))
coldat$Colors <- c(rep('blue',3), rep('red',3),rep('black',3))
coldat$Shapes <- c(rep(19,3),rep(15,3),rep(17,3))

# Check out the metadata table
coldat
```

```
##                   Groups            Genotype Colors Shapes
## AB_1_36hpf            WT                 AB*   blue     19
## AB_3_36hpf            WT                 AB*   blue     19
## AB_2_36hpf            WT                 AB*   blue     19
## sap130aNV_3_36hpf     NV sap130a^pt32a/pt32a    red     15
## sap130aNV_2_36hpf     NV sap130a^pt32a/pt32a    red     15
## sap130aNV_1_36hpf     NV sap130a^pt32a/pt32a    red     15
## sap130aSV_2_36hpf     SV sap130a^pt32a/pt32a  black     17
## sap130aSV_1_36hpf     SV sap130a^pt32a/pt32a  black     17
## sap130aSV_3_36hpf     SV sap130a^pt32a/pt32a  black     17
```

Data exploration before RNAseq analysis

MDS plot before normalization

The multidimensional scaling (MDS) plot, uses a PCA plot constructed from the top genes with largest standard deviations across the samples. These data should provide a relationship among samples, how they cluster together or separate.

```r
# Make sure you have loaded the necessary libraries for the analysis
suppressMessages(library('edgeR'))
library('limma')

# MDS to visualizes the differences between the expression profiles of different
# samples in two dimensions
mds_edger <- plotMDS(x = unique.data_edger,gene.selection = "common",
                     top = nrow(unique.data_edger),
                     col = coldat$Colors, pch = coldat$Shapes,
                     cex = 2.5,
                     main = "Multidimensional Scaling Plot\nbefore normalization")
grid(lty = 6)
legend('bottomleft',legend = c('WT','NV','SV'),
       col =c('blue','red','black'),pch = c(19,15,17),cex = 1)
```

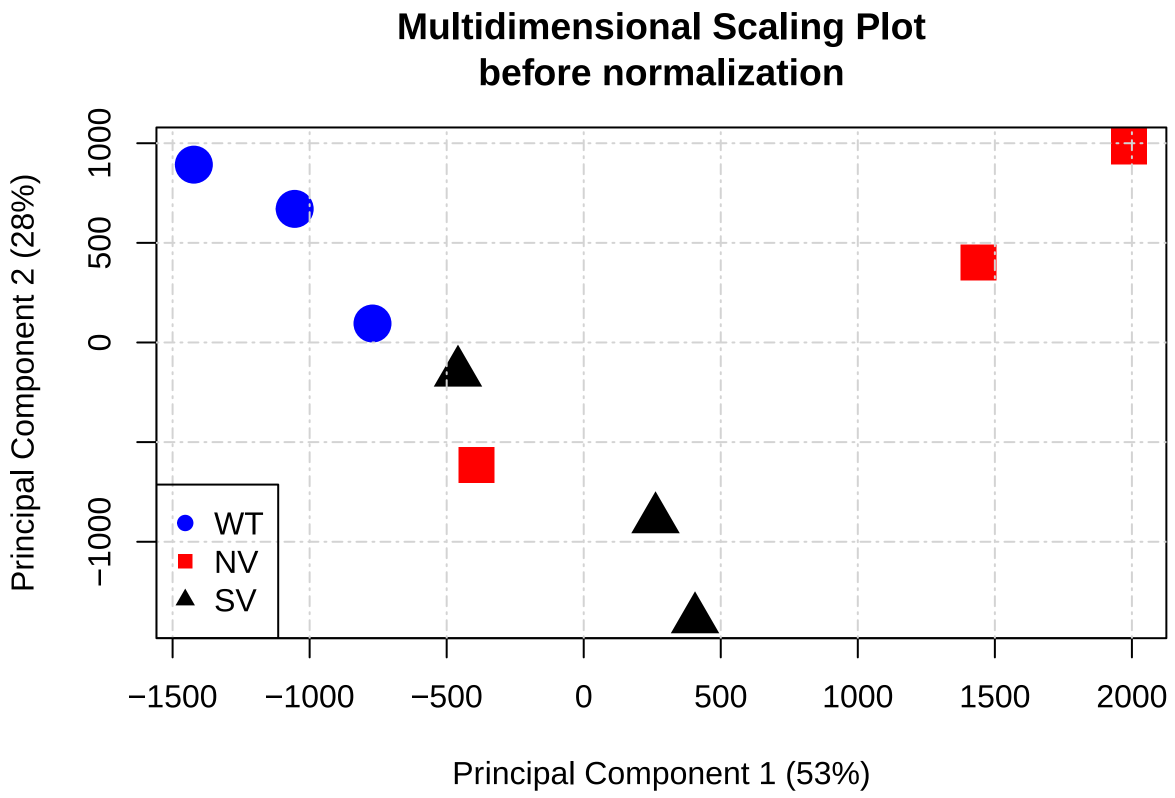

Euclidean distances on the plot approximate the typical log2 fold changes between the samples. Distances on the plot can be interpreted as leading log2-fold-change, meaning the typical (root-mean-square) log2-fold-change between the samples for the genes that distinguish those samples

Making a DGEList data object for edgeR RNAseq analysis

A simple list-based data object used in the edgeR package

```
# Create DGEList object for normalization and differential gene expression analysis
y_edgeR = DGEList(counts=unique.data_edger,
                  genes=row.names(unique.data_edger),
                  samples = coldat,
                  group = coldat$Groups)
```

Filter lowly expressed genes

```
# Default removes all gene rows with less than 15 transcripts across any three
# samples (min.total.count=15) and keeps gene row with 10+ transcripts in more
# than 70% across samples (min.count=10,min.prop=0.7)
keep <- filterByExpr(y_edgeR,group = y_edgeR$samples$Groups)
y_edgeR <- y_edgeR[keep, , keep.lib.sizes=F]
```

Normalize the data

```
y_edgeR <- calcNormFactors(y_edgeR, method = "TMM") # Trimmed Mean of M-values (TMM)
```

What influence did the normalization have on the data

We want to see the samples cluster in their own groups, revealing differences between WT and the *MZsap130a* mutants

```
library("ggplot2")
# Another MDS plot to look at sample grouping
mds_edger2 <- plotMDS(y_edgeR,gene.selection = "common",
                      top = nrow(y_edgeR),
                      col = coldat$Colors,
                      pch = coldat$Shapes,
                      main = "Multidimensional Scaling Plot\nafter normalization",
                      cex = 2.5)+
  theme(axis.title = element_text(size = 30, face = "bold"))
grid(lty = 6)
legend('topleft',legend = c('WT','NV','SV'),
       col =c('blue','red','black'),pch = c(19,15,17),cex = 1)
```

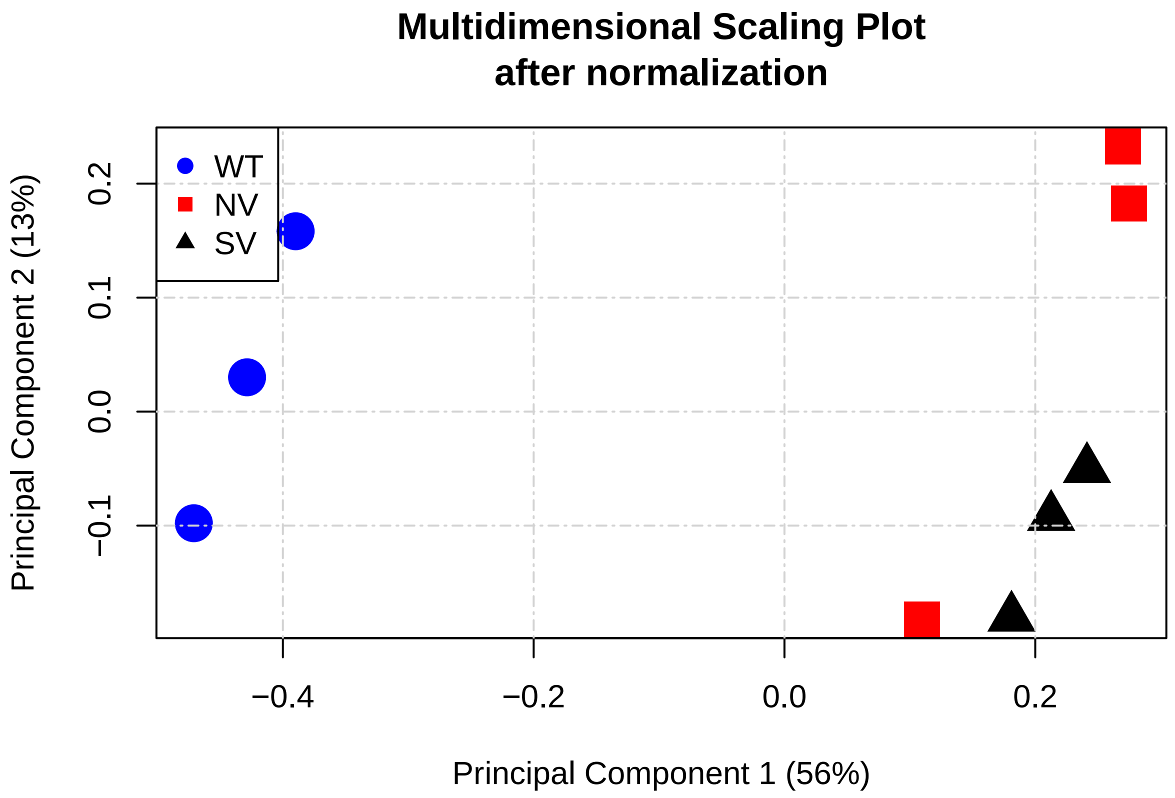
Multidimensional Scaling Plot
after normalization
WT
NV
SV
Principal Component 2 (13%)
0.2
0.1
0.0
−0.1
−0.4
−0.2
0.0
0.2
Principal Component 1 (56%)

Plotting a mean-difference graph to see if the TMM normalization removed composition bias from the libraries

It is important to check the TMM normalization for expected results. So here we look at one sample from each group.

```r
# TMM normalization should have the bulk of genes centered at 0, meaning the
# composition bias from the library sizes has been successfully removed
AB <- edgeR::plotMD.DGEList(y_edgeR, column = 2)+ # Change the sample being plotted
# by changing column = 1-9, for samples 1-9
abline(h=0, col="red", lty=2, lwd=2)
```

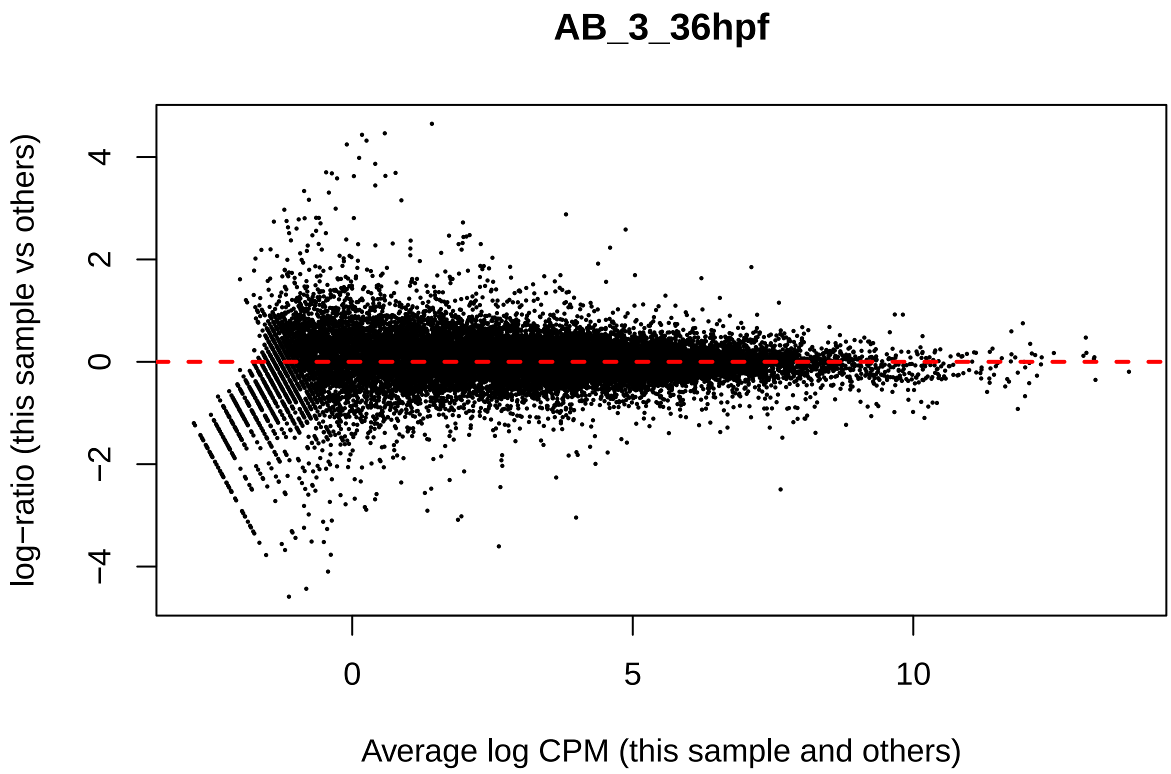


```r
MZsap130a <- edgeR::plotMD.DGEList(y_edgeR, column = 6)+
abline(h=0, col="red", lty=2, lwd=2)
```

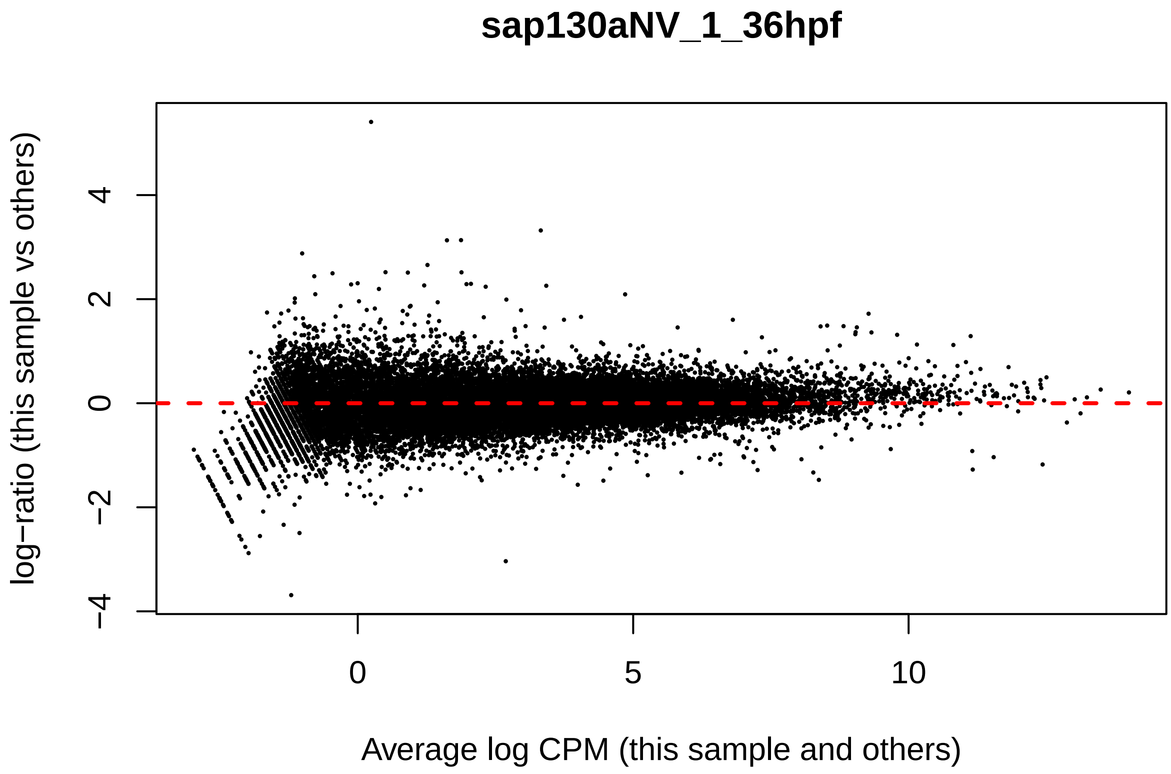


```
MZsap130a <- edgeR::plotMD.DGEList(y_edgeR, column = 9)+
abline(h=0, col="red", lty=2, lwd=2)
```

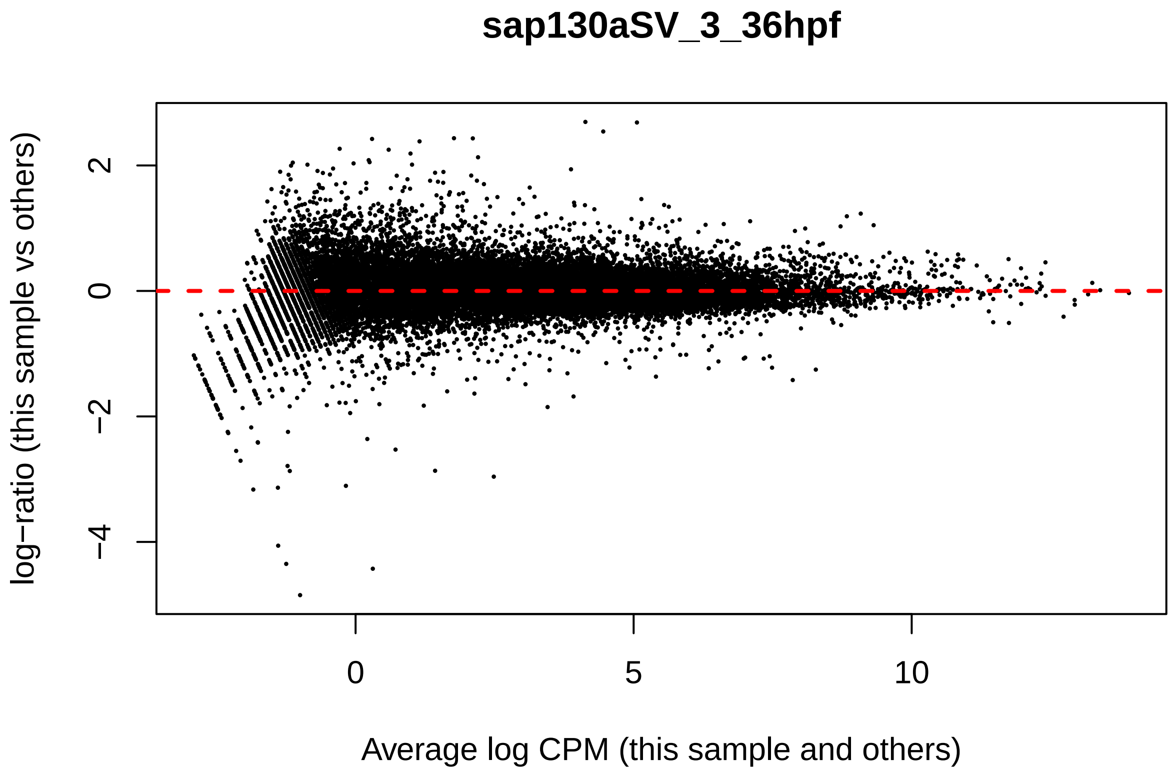

All groups are centered around zero as expected, so the TMM normalization worked well to removed composition bias from the libraries

Creating design matricies to compare WT vs NV vs SV or WT vs *MZsap130a*

```
# Next is the design matrix used to provide info on the samples, in this case the
# groups of samples collected WT, NV, and SV
design_WTvsNVvsSV <- model.matrix(~coldat$Groups)
rownames(design_WTvsNVvsSV) <- rownames(coldat)
colnames(design_WTvsNVvsSV) <- c("WT","NV","SV")

# This design matrix will compare the genotypes
design_WTvsMZsap130a <- model.matrix(~coldat$Genotype)
rownames(design_WTvsMZsap130a) <- rownames(coldat)
colnames(design_WTvsMZsap130a) <- c("WT","MZsap130a")

# The WT data is always the intercept for comparison to NV or SV groups and all
# MZsap130a samples
design_WTvsNVvsSV
```

```
##                    WT NV SV
## AB_1_36hpf          1  0  0
## AB_3_36hpf          1  0  0
## AB_2_36hpf          1  0  0
## sap130aNV_3_36hpf   1  1  0
## sap130aNV_2_36hpf   1  1  0
## sap130aNV_1_36hpf   1  1  0
## sap130aSV_2_36hpf   1  0  1
## sap130aSV_1_36hpf   1  0  1
## sap130aSV_3_36hpf   1  0  1
## attr(,"assign")
## [1] 0 1 1
## attr(,"contrasts")
## attr(,"contrasts")$`coldat$Groups`
## [1] "contr.treatment"
```

```
design_WTvsMZsap130a
```

```
##                    WT MZsap130a
## AB_1_36hpf          1         0
## AB_3_36hpf          1         0
## AB_2_36hpf          1         0
## sap130aNV_3_36hpf   1         1
## sap130aNV_2_36hpf   1         1
## sap130aNV_1_36hpf   1         1
## sap130aSV_2_36hpf   1         1
## sap130aSV_1_36hpf   1         1
## sap130aSV_3_36hpf   1         1
## attr(,"assign")
## [1] 0 1
## attr(,"contrasts")
## attr(,"contrasts")$`coldat$Genotype`
## [1] "contr.treatment"
```

Here I wanted to search for differences between the NV and SV groups when compared to WT, to accomplish

this edgeR implements an ANOVA-like analysis across the samples. I hoped to find differences that could explain the difference in phenotype seen among the *MZsap130a* mutants.

Estimate the dispersions among genes and samples comparing WTvsNVvsSV

```
# Estimate dispersions
# Common dispersion = the mean dispersion across all genes
y_edger = estimateGLMCommonDisp(y_edgeR,design_WTvsNVvsSV,verbose = T)
```

```
## Disp = 0.02129 , BCV = 0.1459
```

```
# Trended dispersion = the mean dispersion across all genes with similar abundances
# In other words, the fitted value of the mean-dispersion trend.
y_edger = estimateGLMTrendedDisp(y_edger,design_WTvsNVvsSV)

# Tagwise dispersion = the gene specific dispersions, so across samples
y_edger = estimateGLMTagwiseDisp(y_edger,design_WTvsNVvsSV)
```

Plotting the biological coefficient of variation for WTvsNVvsSV

This allows for visualization of the biological variance you wish to study. It represents the covariate of variation that would remain between biological replicates if sequencing depth could be increased indefinitely. For human cancer RNAseq data a BCV around 0.40 is common but with isogeneic mouse lines this drops to 0.10, anything between these values is a reasonable dataset to explore further.

```
# Make a variable to hold the Biological coefficient of variation (BCV)
bcv <- sqrt(as.numeric(y_edger[["common.dispersion"]]))

# plots the tagwise biological coefficient of variation (square root of dispersions)
# against log2-CPM
plotBCV(y_edger, xlab = "Average Log2CPM")
text(x = 11,y =0.4, labels = round(bcv, digits = 5))
```

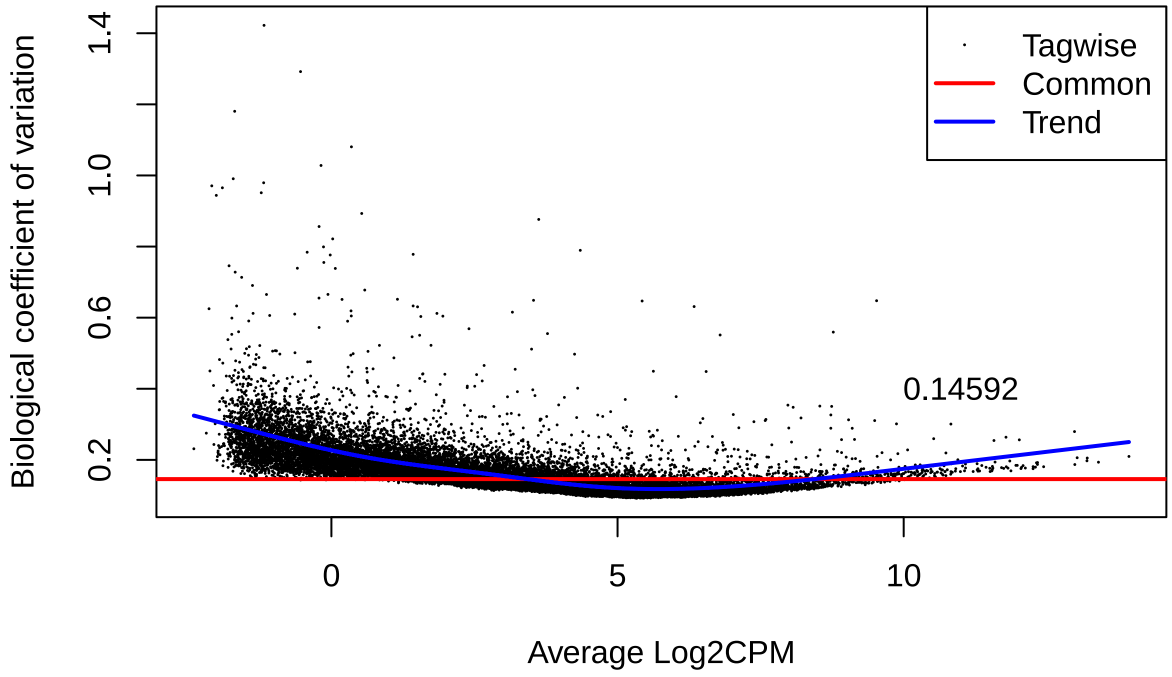

Estimate the dispersions among genes and samples comparing WT vs *MZsap130a*

```
# Estimate dispersions
# Common dispersion = the mean dispersion across all genes
y_edgerMZ = estimateGLMCommonDisp(y_edgeR,design_WTvsMZsap130a,verbose = T)
```

```
## Disp = 0.02307 , BCV = 0.1519
```

```
# Trended dispersion = the mean dispersion across all genes with similar abundances.
# In other words, the fitted value of the mean-dispersion trend.
y_edgerMZ = estimateGLMTrendedDisp(y_edgerMZ,design_WTvsMZsap130a)

# Tagwise dispersion = the gene specific dispersions, across samples
y_edgerMZ = estimateGLMTagwiseDisp(y_edgerMZ,design_WTvsMZsap130a)
```

Plotting the biological coefficient of variation for WT vs *MZsap130a*

Noticed a slight increase in BCV, suggesting combining the NV and SV groups into one MZsap130a group, reveals more differences from WTs

```
# Make a variable to hold the Biological coefficient of variation (BCV)
bcv <- sqrt(as.numeric(y_edgerMZ[["common.dispersion"]]))
```

```r
# The tagwise biological coefficient of variation (square root of common dispersion)
# against log2-CPM
plotBCV(y_edgerMZ, xlab = "Average Log2CPM")
text(x = 11,y =0.4, labels = round(bcv, digits = 5))
```

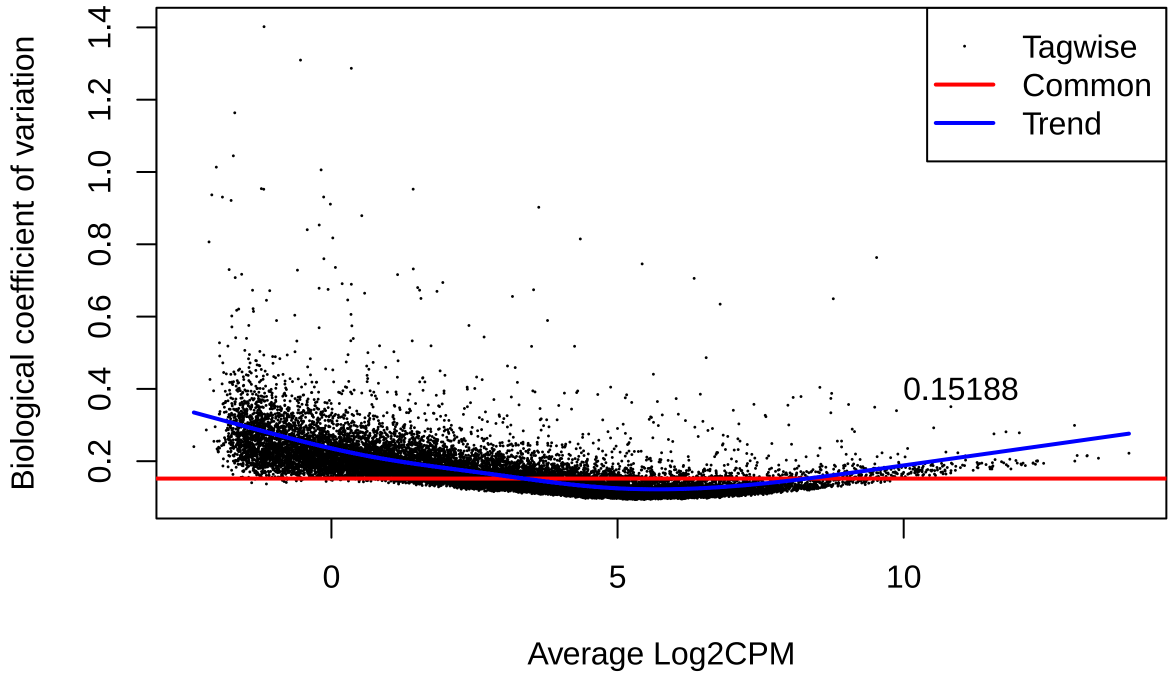

Now we fit the model using glmQLFit()

```r
# Fit to the model comparing WTvsNVvsSV
fit_edger = edgeR::glmQLFit(y_edger, design = design_WTvsNVvsSV)

# Fit to the model comparing WTvsMZsap130a
fit_edgerMZ = edgeR::glmQLFit(y_edger, design = design_WTvsMZsap130a)
```

glmQLFit() uses the trended NB dispersion to fit a Generalized Linear Model (GLM), and then estimates the Quasi-Likelihood (QL) dispersion from the deviance. It doesn't re-estimate the NB dispersion but instead estimates an entirely different QL dispersion for each gene. In contrast, glmFit() uses the tagwise NB dispersion to fit a GLM - and that's it.

Finally the analysis using glmQFLTest() and topTags() on the fitted data from the previous step

```
# The analysis for WT vs NV vs SV, ANOVA-like edgeR
edgeR.results_ql = as.data.frame(edgeR::topTags(
  edgeR::glmQLFTest(glmfit = fit_edger, coef = c(2:3)),n=nrow(unique.data_edger)))

# The analysis for WT vs MZsap130a, ANOVA-like edgeR
edgeR.results_qlMZ = as.data.frame(edgeR::topTags(
  edgeR::glmQLFTest(glmfit = fit_edgerMZ),n=nrow(unique.data_edger)))
```

Do *MZsap130a* NV and SV groups have transcript differences that can explain their physical differences

Here I looked at the genes that had opposite trends in the NVs and the SVs. This revealed some genes that have not been identified or studied in zebrafish heart development.

```
# Keeps significant genes with FDR<=0.05 and
#abs(log2FC) >= 0.4 (which = 1.3+ or - fold change)
Sig_edgeR_DEGs <- edgeR.results_ql[
  which(edgeR.results_ql$FDR<=0.05 & abs(edgeR.results_ql$logFC.SV)>=0.4),]

Sig_edgeR_DEG_NV <- edgeR.results_ql[
  which(edgeR.results_ql$FDR<=0.05 & abs(edgeR.results_ql$logFC.NV)>=0.4),]

# Genes that are significant in WT vs NV and not WT vs SV
sig_inNV_notSV <- Sig_edgeR_DEG_NV[
  -(which(Sig_edgeR_DEG_NV$genes %in% Sig_edgeR_DEGs$genes)),]

# Keep genes with a difference in log2FC of 0.6
sig_diff_NV <- sig_inNV_notSV[
  which((sig_inNV_notSV$logFC.NV-sig_inNV_notSV$logFC.SV)>=0.6),]

# Genes that are significant in WT vs SV and not WT vs NV
sig_inSV_notNV <- Sig_edgeR_DEGs[
  -(which(Sig_edgeR_DEG_NV$genes %in% Sig_edgeR_DEGs$genes)),]

sig_diff_SV <- sig_inSV_notNV[
  which((sig_inSV_notNV$logFC.NV-sig_inSV_notNV$logFC.SV)>=0.6),]

dim(sig_diff_NV) # Only six genes showing large 0.6 difference
```

```
## [1] 6 7
```

```
dim(sig_diff_SV) # 22 genes or rows
```

```
## [1] 22  7
```

Basic RNAseq Plots to Explore the Data

Volcano plots

Libraries needed for volcano plots

```
# Libraries for plotting data
library('ggplot2')

# Load packages
suppressMessages(library('tidyverse'))
library('ggrepel')
```

Data preparation for volcano plots

```
volcano_datSV <- data.frame (GeneNames = rownames(edgeR.results_ql),
                             log2FC = edgeR.results_ql$logFC.SV,
                             FDR = edgeR.results_ql$FDR)

rownames(volcano_datSV) <- volcano_datSV$GeneNames

# making a column that labels who is in which group for plotting
volcano_datSV <- volcano_datSV %>%
  mutate(
    Significance = case_when(
      abs(log2FC) >= 0.4 & FDR < 0.05 ~ "log2FC & FDR",
      abs(log2FC) <= 0.4 & FDR < 0.05 ~ "FDR",
      abs(log2FC) >= 0.4 & FDR > 0.05 ~ "log2FC",
      abs(log2FC) <= 0.4 & FDR > 0.05 ~ "NS")
  )
```

Lets make another for WT vs NV

```
volcano_datNV <- data.frame (GeneNames = rownames(edgeR.results_ql),
                             log2FC = edgeR.results_ql$logFC.NV,
                             FDR = edgeR.results_ql$FDR)

rownames(volcano_datNV) <- volcano_datNV$GeneNames

#making a column that labels who is in which group for plotting
volcano_datNV <- volcano_datNV %>%
  mutate(
    Significance = case_when(
      abs(log2FC) >= 0.4 & FDR < 0.05 ~ "log2FC & FDR",
      abs(log2FC) <= 0.4 & FDR < 0.05 ~ "FDR",
      abs(log2FC) >= 0.4 & FDR > 0.05 ~ "log2FC",
      abs(log2FC) <= 0.4 & FDR > 0.05 ~ "NS")
  )
```

Selected genes for labels in the volcano plot

```
# Selected genenames for volcano plots
vol_cellComm_Sarcomere <- c("jph2","jph1b","sgca","cxcr4b",
                            "actc1","ttn.1","ttn.2","acta1a")

vol_sarco_cellCycle <-c("actc1","ttn.1","ttn.2",
                        "e2f7","brca2","vrk1","haus6")

vol_Mito_metabo <- c("mtrf1","mrpl58","slc25a3a",
                     "acox","acsl1a", #fatty acid
                     "ugpa2a","phkb") #glycogen metabo.

vol_OFT_SmoMuscle <- c("isl1",
                       "tagln2","cnn2")
```

Overlaping genes found from DEG analyis of *sap130b* vs WT and *sap130a* vs WT at 36hpf whole embryos

```
# List for the overlap DEGs in MZsap130a vs MZsap130b at 36hpf whole embryo
overlap <- c("hbbe2","hbbe3","hebp2","hbae3","hbae5", # 1st row hemoglobins
             "cyp4v2a","cyp1b1","cyp46a1.1","cyp2x6") # 2nd row is cytochromes
# associated with heme, iron, oxygen binding

sarco <- c("actc1","ttn.1","ttn.2","myh6","myl4","cacng1a","myh7","tpm4a","tcap","tnnt2a")

newnew <- c("actc1","cxcr4b","myh6","myh7","myl7","myzap","ttn.1","gja3","myl4")
```

Set up the theme for our volcano plot, very powerful tool for consistant plotting

```
# Theme set up to control all elements
mi_teme <- theme(title = element_text(size = 14, face = "bold"),
                 plot.title.position = "panel",
                 plot.title = element_text(vjust =-2,hjust = 0.5),
                 axis.text.x = element_text(size= 10),
                 axis.text.y = element_text(size = 10),
                 axis.title.x = element_text(face = "bold",size = 12),
                 axis.title.y = element_text(face = "bold",size = 12),
                 legend.text = element_text(size = 10, face = "bold"),
                 legend.title = element_blank(),
                 legend.position = "top",
                 legend.spacing.x = unit(0.2,"picas"),
                 legend.key = element_blank(),
                 legend.box.spacing = unit(0.001,"picas"),
                 legend.key.size = unit(0.001,"picas"),
                 plot.margin = unit(c(0.01,1,0.01,1),"mm"))
```

Make a volcano plot to visualize all the data at once

Here I plot all the genes in one graph with cuts offs set to an FDR <0.05 and a log2FC > 0.4 (~1.3logFC). Looking for heart specific changes with whole embryo resolution we wanted to include lower log2FC values, to increase the odds of finding changes we know are important to heart development.

```
# Make a list of desired genes to plot
s <- volcano_datSV %>%
  filter(GeneNames %in% vol_Mito_metabo)

# Make a ggplot volcano, similar to EnhancedVolcano
# title etc
volcanoMan <- ggplot(volcano_datSV, aes(log2FC,-log(FDR,10)))+
  geom_point(aes(color=Significance),size = 0.5, alpha = 4/5)+
  scale_color_manual(values = c("NS"="darkgrey","log2FC & FDR"="seagreen",
                                "log2FC"="orangered3","FDR"="lightblue"))+
  xlab(bquote(~Log[2]~fold~change))+ xlim(-5,5)+
  ylab(bquote(~-Log[10]~italic(FDR)))+ylim(0,6)+
  geom_hline(yintercept = -log10(0.05))+
  geom_vline(xintercept = c(-0.4,0.4))+
  ggtitle(expression(bolditalic("Tg(myl7:EGFP)")~bold("vs")~
                       bolditalic("MZsap130a;Tg(myl7:EGFP)"~bold("SV"))))+
  geom_label_repel(data = s,mapping = aes(label = GeneNames), size = 4,
                   force = 0.5,direction = "both",
                   nudge_y = c(rep(0.35,5),rep(-.25,2),rep(0.35,3)),
                   arrow = arrow(length = unit(0.01, 'npc'),ends = "last",type = "closed"),
                   segment.color = 'black',segment.size = 0.4,max.iter = 3e3,
                   min.segment.length = 0, seed = 42,
                   fontface = "bold.italic",label.padding = 0.1)+
  geom_point(data =s ,color = "red",size=1)+
  guides(color= guide_legend(reverse = TRUE, override.aes = list(size=5)))

suppressWarnings(print(volcanoMan + mi_teme))
```

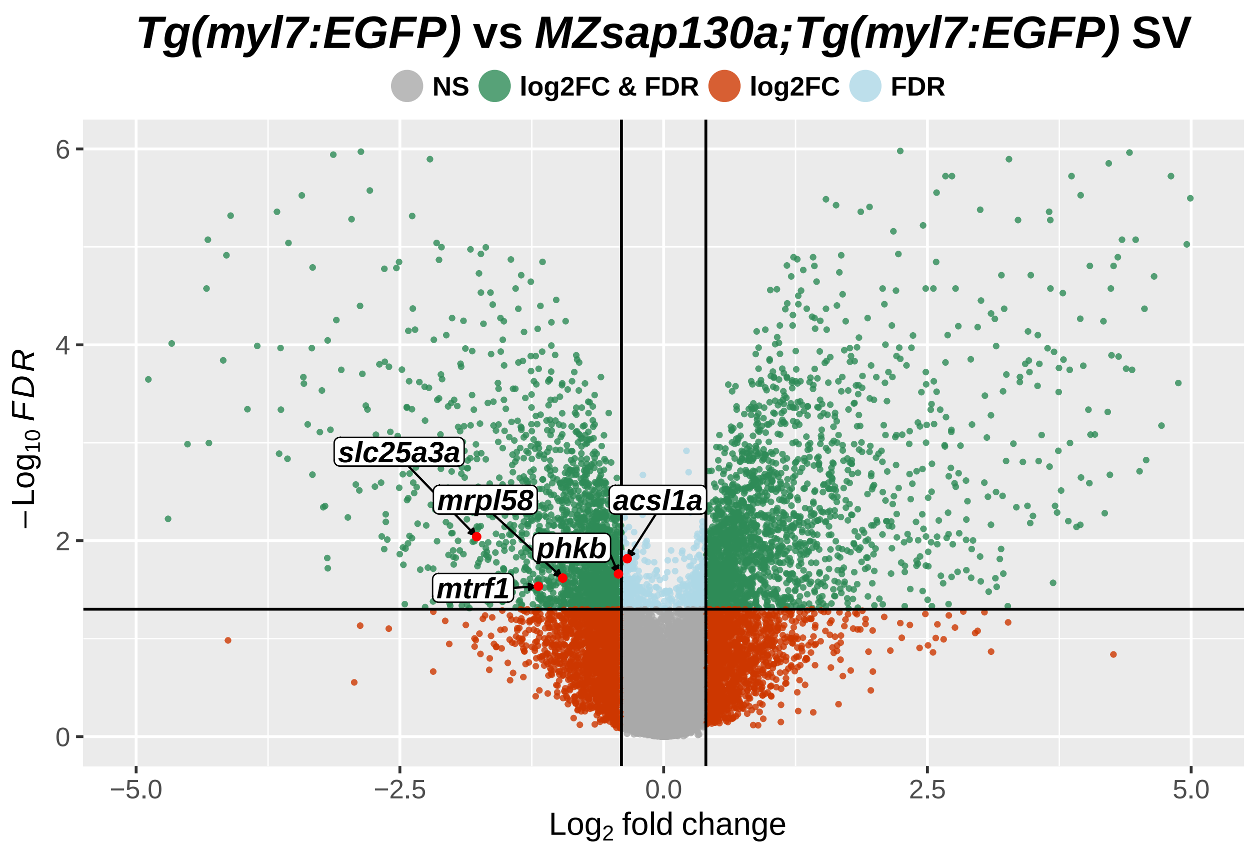

Taking a look at the WT vs NV data

```
# Make a list of desired genes to plot
s <- volcano_datNV %>%
  filter(GeneNames %in% vol_Mito_metabo)

# Make a ggplot volcano, similar to EnhancedVolcano
# title etc
volcanoMan <- ggplot(volcano_datNV, aes(log2FC,-log(FDR,10)))+
  geom_point(aes(color=Significance),size = 0.5, alpha = 4/5)+
  scale_color_manual(values = c("NS"="darkgrey","log2FC & FDR"="seagreen",
                                "log2FC"="orangered3","FDR"="lightblue"))+
  xlab(bquote(~Log[2]~fold~change))+ xlim(-5,5)+
  ylab(bquote(~-Log[10]~italic(FDR)))+ylim(0,6)+
  geom_hline(yintercept = -log10(0.05))+
  geom_vline(xintercept = c(-0.4,0.4))+
  ggtitle(expression(bolditalic("Tg(myl7:EGFP)")~bold("vs")~
                     bolditalic("MZsap130a;Tg(myl7:EGFP)"~bold("NV"))))+
  geom_label_repel(data = s,mapping = aes(label = GeneNames), size = 4,
                   force = 0.5,direction = "both",
                   nudge_y = c(rep(0.35,5),rep(-.25,2),rep(0.35,3)),
                   arrow = arrow(length = unit(0.01, 'npc'),ends = "last",type = "closed"),
                   segment.color = 'black',segment.size = 0.4,max.iter = 3e3,
                   min.segment.length = 0, seed = 42,
```

```
                    fontface = "bold.italic",label.padding = 0.1)+
  geom_point(data =s ,color = "red",size=0.6)+
  guides(color= guide_legend(reverse = TRUE, override.aes = list(size=2.5)))

suppressWarnings(print(volcanoMan + mi_teme))
```

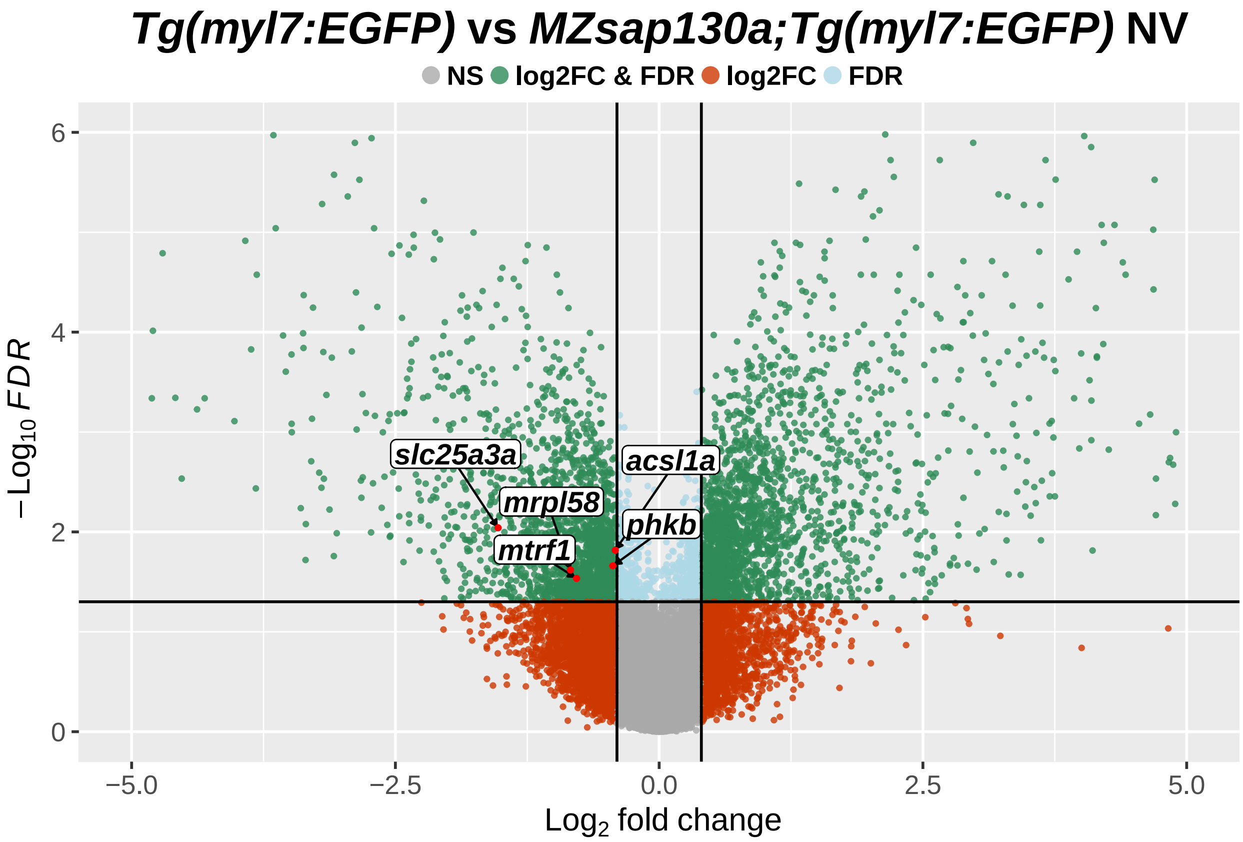

Summary

This report summarizes the basic pipeline used to analyze the *MZsap130a* and *MZsap130b* whole embryo RNAseq data sets. All plots for the publication were made using ggplot2, heatmap3, viridisLite, reshape2, dendextend, tidyverse, and ggrepel. The data is explored and analyzed here based on the edgeR user's guide found here at Bioconductor

WT vs *MZsap130a* Hearts 48hpf

Ricardo A. DeMoya

2023-06-20

RNAseq analysis

The csv file that contains the count data to be uploaded, is from total RNA collected from isolated hearts at 48hpf for WT and *MZsap130a* genotypes. The total RNA was sent to sequencing core at the University of Pittsburgh with one pooled biological replicate for each group (WT1, *MZsap130a*, ~150 hearts per sample). Once the fastq files were obtain we uploaded them in CLC Genomics Workbench by QIAGEN and used the RNAseq analysis tool to produce a Total count matrix for export as a csv file. This file was then used in R and Rstudio to explore the relationship between WT and *MZsap130a* mutant hearts at 48hpf.

Read in the count matrix

Using the read.table() function, we import the data table from a csv count matrix generated by CLC Gx workbench. Other alignment methods can be used including, Rbowtie2, TopHat2, Rsubread, or HISAT2. Annotation was done using GRCz11 zebrafish reference genome.

```
data_edger = read.table(
  file="data/processed_data/MZsap130a_vs_crtls_hearts_only2.csv",
  sep=",",
  header=T)
PseudoTech<-read.table(
  file ="data/processed_data/MZsap130a_vs_crtls_hearts_only3_pseudoTechreps.csv",
  header = T,
  sep = ",")
```

Clean up the data and prepare for edgeR RNAseq analysis

```
#Updating some genenames
data_edger$Name[data_edger$Name == "ACTC1"] <- "actc1"

#for smoMuscle OFT SHF ltbp3
data_edger$Name[data_edger$Name == "FP102018.1"] <- "ltbp3"
```

Genenames and IDs for later use in plotting

```
data_IDsandNames <- data_edger[,1:2]
data_IDsandNames[2] <- gsub("https://www.ensembl.org/id/","",data_IDsandNames$Identifier)

data_edger[2] <- gsub("https://www.ensembl.org/id/","",data_IDsandNames$Identifier)

# this will make the list a data frame
data_edger <- data.frame(data_edger[,2:4], row.names = data_edger$Name)

pseudo <- data.frame(PseudoTech[,3:8], row.names = PseudoTech[,1])
```

```
#cnts will contain the ensemble ID or gene name
cnts <- data_edger
#strip away the ensembel website info leaving just the ID
cnts[1] <- gsub("https://www.ensembl.org/id/","",cnts$Identifier)

#Make a clean count dataframe with rownames as genes and colnames as samples
cnts2 <- data.frame(WT = cnts[,2], MZsap130a = cnts[,3], row.names = rownames(cnts))

head(cnts[,1:3], n=5)
```

```
##                    Identifier WT_S13..GE....Total.counts
## rpl24      ENSDARG00000099104                      12675
## cep97      ENSDARG00000102407                        350
## nfkbiz     ENSDARG00000102097                        120
## CU651657.1 ENSDARG00000099319                          3
## eed        ENSDARG00000099640                        476
##            MZsap130a_pt32a_S14..GE....Total.counts
## rpl24                                         6737
## cep97                                          199
## nfkbiz                                          45
## CU651657.1                                       2
## eed                                            219
```

Check for replicates

```
#This makes a list of ENSEMBL IDs in the data set
unique_edger = names(which(table(as.vector(cnts[,1])) == 1))
#This indexes your data for rows that are present in the list you made in the last step
unique.data_edger = cnts[which(cnts[,1] %in% unique_edger),]
#This makes sure that your data is in a dataframe format
unique.data_edger = data.frame(WT = unique.data_edger[,2],
                               MZsap130a = unique.data_edger[,3],
                               row.names = rownames(unique.data_edger))
#This will remove the ID and names columns in this data set column 1 and 2
unique.data_edger = unique.data_edger[,-1]
```

Create a metadata table

```
# Metadata for modeling the sequencing experiment
coldat <- data.frame(row.names = colnames(cnts2),stringsAsFactors = T)
coldat$Genotype <- factor(c("WT","MZsap130a"),
                          levels = c("WT","MZsap130a"))
coldat$Colors <- c('blue','pink')
coldat$Shapes <- c(19,14)

# Metadata for modeling the sequencing experiment
coldat2 <- data.frame(row.names = colnames(pseudo),stringsAsFactors = T)
coldat2$Genotype <- factor(c(rep("WT",3),rep("MZsap130a",3)),
                           levels = c("WT","MZsap130a"))
coldat2$Colors <- c(rep('blue',3),rep('pink',3))
coldat2$Shapes <- c(rep(19,3),rep(14,3))
```

RNAseq analysis with no replicates

The edgeR user's guide suggest to use a likelihood ratio test to check for trends in datasets without replicates.

```
suppressMessages(library("edgeR"))
```

edgeR Likelihood ratio test

```
# Create DGEList object
y_edgeR = DGEList(counts=cnts2,
                  genes=row.names(cnts2),
                  samples = coldat,
                  group = coldat$Genotype)
```

Filter lowly expressed genes

Removes all gene rows with less than 15 transcripts in all samples (min.total.count=15) and gene expresses 10+ transcripts in more than 70% across samples (min.count=10,min.prop=0.7)

```
keep <- filterByExpr(y_edgeR,group = y_edgeR$samples$Genotype)
y_edgeR <- y_edgeR[keep, , keep.lib.sizes=F]
```

Manual estimate of BCV

This is the second option proposed by the edgeR manual for datasets without replicates

```
#The BCV calculated for the other whole embryo experiments range between 0.14-0.21
bv <- 0.2
exc <- exactTest(y_edgeR,dispersion = bv^2)

exc_Resu <- data.frame(exc[["table"]], row.names = rownames(exc))
exc_Resu[,4] <- rownames(exc)
```

Normalize the data

Using Relative Log Expression, scaling by a factor geometric mean of each sample over the median library for that sample

```
y_edgeR <- calcNormFactors(y_edgeR, method = "RLE")

norm_cnts <- data.frame(cpm(y_edgeR, normalized.lib.sizes = F,log = T))
norm_cnts2 <- norm_cnts
norm_cnts2$Genes <- rownames(norm_cnts2)
```

Next is the design matrix used to provide info on the samples

```
design_edger <- model.matrix(~coldat$Genotype)
```

No dispersion without replicates

```
# Fit model coldat
fit = glmFit(y_edgeR,design = design_edger,dispersion = bv^2)

lrt <- glmLRT(fit)
```

Get the output from lrt()

```
lrt_output <- data.frame(lrt[["table"]],row.names = rownames(lrt))
lrt_output[,5] <- rownames(lrt_output)
```

```
Up_lrt <- lrt_output[which(lrt_output$logFC>=0.5 & lrt_output$PValue<=0.1),]
Dwn_lrt <- lrt_output[lrt_output$logFC<=-0.5 & lrt_output$PValue<=0.1,]
```

Mean difference plot

The genes that are up or down 2 fold change are colored purple or orange, respectively.

```
plotMD(lrt, main = "Differentially Expressed Genes\n48hpf WT vs MZsap130a hearts",
       hl.pch = 2,hl.col = c("purple","darkorange"))
abline(h=c(-1, 1),col="black", v = 2)
```

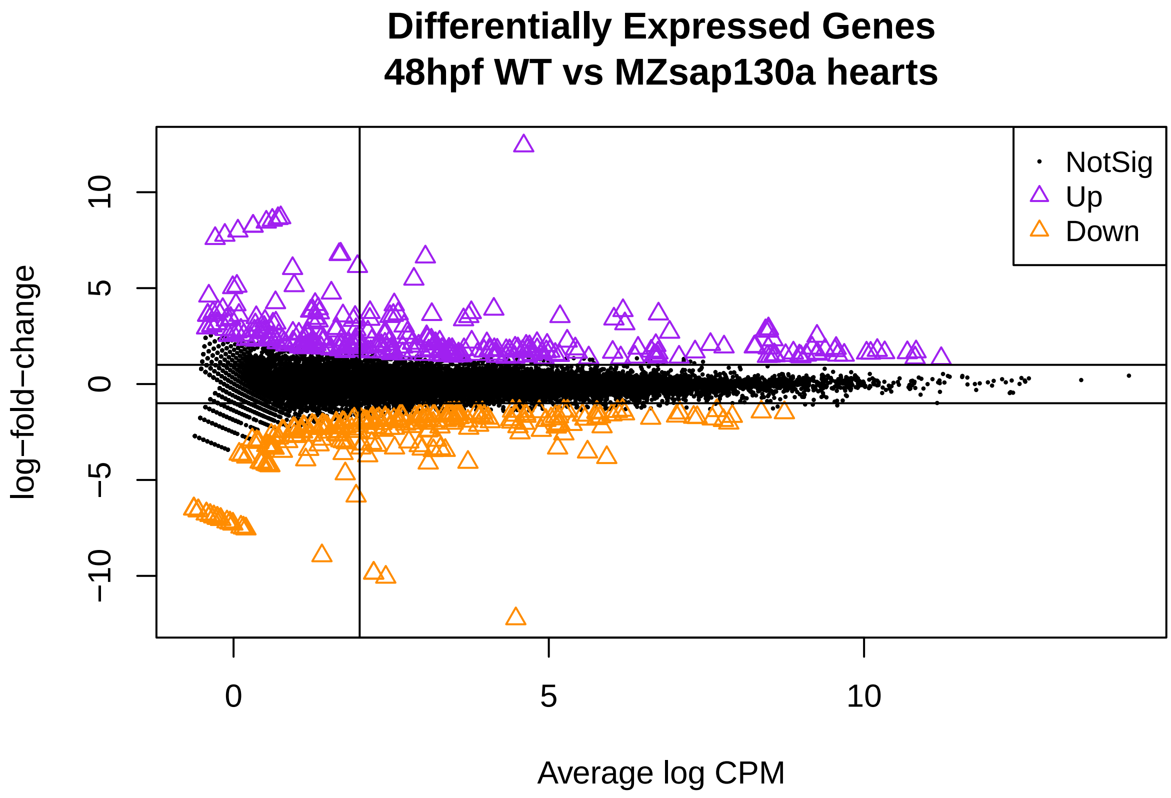

Plotting a heatmap

Libraries for plotting

```
library('ggplot2')
library('heatmap3')
library("viridisLite")
suppressMessages(library("reshape2"))
suppressMessages(library("dendextend"))
```

Make a function to italicize genenames

```
make_italics <- function(x) {
  as.expression(ifelse(is.na(x)==F ,lapply(x, function(y) bquote(italic(.(y)))),NA))
}
```

```
new_one <- c("vrk1","mcts1","ccne1","dscc1","usp37","mcm5","e2f7","haus6","cdkn1a","rb1",
             "gadd45bb", # cell cycle
             "ttn.1","ttn.2","actc1","myh7","myh6","myl7","kcnk3b","scn4aa","scn4ba",
             "scn2b","cacnb1","cacna1sb","cacng7a") # sarcomere

# Cell junction and dystrophin glycoprotein complex
# Connects the ECM and muscle cells in heart and skeletal
cell_comm <- c("gja3a","gja8b","cxcr4b","cxcl12a","cxcl12b","dag1","dagla","cav1","cav2",
               "cav3","dtna","snta1","sntb1","sntg2","sntb2","sgcg","sgca","dnm3a",
               "dnm2a","dnm2b","dmnla","dnmlb","tln1","tln2b","vcla","vclb","desma",
               "desmb","jph1a","jph1b","jph2","jph3","cldn2","cldn15la","cldn11a",
               "cldn11b","cldn12")
```

Genes for heatmaps of groups selected

```
heat_cellComm_Sarcomere <- c("gja3a","gja8b","cxcr4b","cxcl12a","cxcl12b","dag1",
                             "dagla","cav1","cav2","cav3","dtna","snta1","sntb1",
                             "sntg2","sntb2","sgcg","sgca","dnm3a","dnm2a","dnm2b",
                             "dmnla","dnmlb","tln1","tln2b","vcla","vclb","desma",
                             "desmb","jph1a","jph1b","jph2","jph3","cldn2","cldn15la",
                             "cldn11a","cldn11b","cldn12","ttn.1","ttn.2","actc1",
                             "kcnk3b","kcnk2b","scn4aa","scn4ba","scn2b","cacna1bb",
                             "cacnb1","cacna1sb","cacng7a")

heat_sarco_cellCycle <-c("ttn.1","ttn.2","actc1","scn4aa","scn4ba","scn2b","cacna1bb",
                         "cacnb1","cacna1sb","cacng7a","myh7","myh6","myl7","kcnk3b",
                         "vrk1","ccne1","cdkn1a","dscc1","usp37", "e2f7","gadd45bb",
                         "haus6","mcm5","mcts1","rb1")

heat_Mito_metabo <- c("dnm1a","dmn3a","mtrf1","mtrf1l","mrpl58","slc25a15b","slc25a44a",
                      "slc25a42","slc25a3a",
                      "acox3","ppt2","mecr", #fatty acid
```

```
                      "ugp2a","hdac8","phka2") #glycogen metabolism

heat_OFT_SmoMuscle <- c("tagln","tagln2","acta2","cnn2","myh11a","acta1a","myh11b",
                        "mef2cb","mef2ca","ltbp3","isl1","isl2a","isl2b")


mitoMito <- c("micall2a","ndufaf6","acox3","acoxl","cox14","cox15","mrpl2","mrpl9",
              "mrpl14","mrpl19","mrpl39","mrpl58","mrps2","dnm1a","dmn3a","mtrf1",
              "mtrf1l","mrpl58","slc25a15b","slc25a44a","slc25a42","slc25a3a",
              "acox3","ppt2","mecr", #fatty acid
              "ugp2a","hdac8","phka2") #glycogen metabo
```

Prepare data for heatmap

```
# Filter the results by FDR
de.genes_edger = norm_cnts[which(rownames(norm_cnts) %in% heat_OFT_SmoMuscle),1:2]

# Alphabetic order for rownames
sort_de.genes <- de.genes_edger[order(rownames(de.genes_edger)),]

# scales by standard deviations but already normalized for library size in cpm
scaled <- scale(sort_de.genes, scale = T)
hr_edger = hclust(dist(scaled, method="euclidean"), method="average")
hc_edger = hclust(dist(t(scaled), method="euclidean"), method="average")
```

```
#Set colors for heatmap labels WT blue, MZsap130a black
colors2 = c('blue','black')

labz <- make_italics(rownames(scaled))

cellCommNames <- c("vclb","ttn.1","sgcg","scn2b","jph2","gja8b","desma",
                   "cxcr4b","cldn2","cav2","cacna1sb","actc1")
cellCycNames <- c("vrk1","ttn.2","scn4ba","rb1","myh7","mcts1","haus6",
                  "e2f7","cdkn1a","cacng7a","cacna1sb","actc1")
```

```
#Creates a list of names without the other genes crowding the plot
labels_me <- rownames(scaled)
labels_me[!labels_me %in% heat_OFT_SmoMuscle] <- NA
labs <- make_italics(labels_me)
```

Make a heatmap

```
heatmap_edger = heatmap3::heatmap3(x = scaled,
          Colv =NA,
          Rowv =NA,
          col=viridis(256),
          margins = c(0.25,5),
          labRow=labz,
          cexRow = 0.8,
```

```
           ColSideColors = colors2, labCol = "" ,
           legendfun = function() heatmap3::showLegend(legend = c(expression(bold("Up")),
           expression(bold("Down"))),
           col = c("yellow","purple4"),
           lwd = 10,cex = 1, inset = c(0.2,0.1)),
           ColSideLabs = "")

par(lend = 1)
legend("bottom",inset = c(0,1),
       legend =  c(expression(bolditalic("Tg(myl7:EGFP)")),
                   expression(bolditalic("MZsap130a;Tg(myl7:EGFP)"))),
       col = c('blue', 'black'),
       lty = 1,##type of legend label 1=solid bar, 2= dashed line, etc
       lwd = 6,##width of legend label bar
       text.font = 1,cex = 0.8,xpd = T,bty = "n")
```

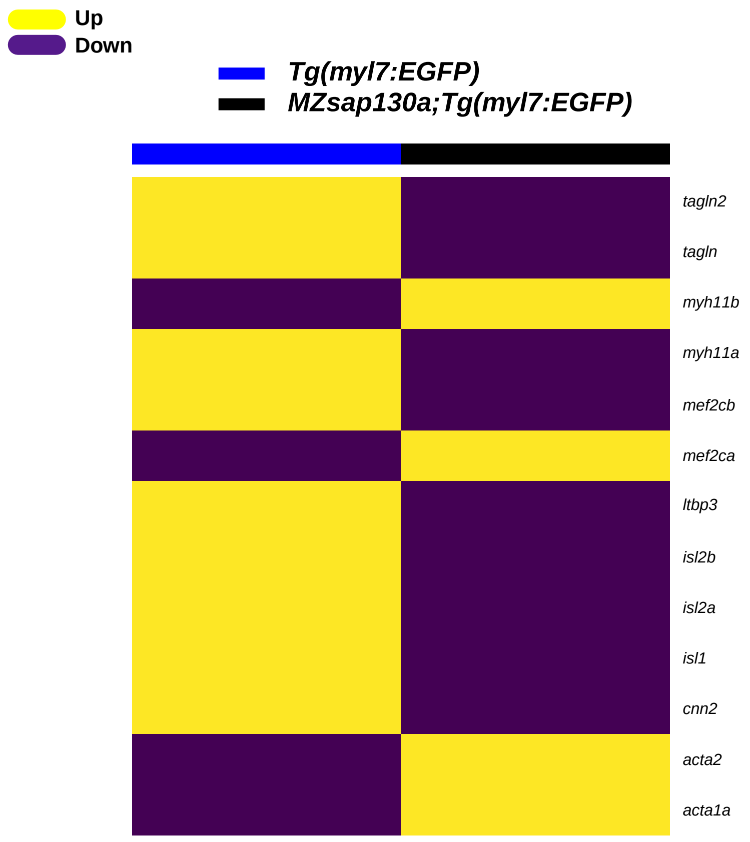

Supplement: Supplementary file 6 [file DataSheet1.PDF]
